# What went right during the COVID crisis: The capabilities of local actors and lasting innovations in oncology care and research

**DOI:** 10.1371/journal.pgph.0002366

**Published:** 2023-09-25

**Authors:** Brenda Bogaert, Zisis Kozlakidis, Elodie Caboux, Julien Péron, Pierre Saintingy

**Affiliations:** 1 Department of Social Sciences and Humanities, Centre Léon Bérard, Lyon, France and Institut des Humanités en Médecine, UNIL/CHUV, Lausanne, Switzerland; 2 International Agency for Research on Cancer, World Health Organization, Lyon, France; 3 Department of Medical Oncology and Research Center on Healthcare Performance (RESHAPE) INSERM U1290, Hospices Civils de Lyon, Pierre-Benite, France; 4 Department of Medical Oncology, Centre Léon Bérard and Claude Bernard Lyon 1 University, INSERM 1052, CNRS 5286, Cancer Research Center of Lyon, Lyon, France; PLOS: Public Library of Science, UNITED STATES

## Abstract

This article will elaborate how oncology care and research was adapted during the COVID pandemic in the Metropole of Lyon (France), including the lasting innovations that came out of the crisis. The research method involved 22 semi-structured qualitative interviews of healthcare professionals, managers, and researchers in the Lyon, France region coming from both public and private academic hospitals. The interviews took place from February 2021-December 2022 in order to assess the long-term adaptations and innovations in cancer care organization in the post-COVID era. The main results show adaptations and innovations in 1) new processes and resources to facilitate disciplinary and interdisciplinary work; 2) harmonization and streamlining of patient journeys. In the discussion section, we will mobilize the capabilities approach, an interdisciplinary social sciences approach that focuses on the capabilities of persons to *be* and to *do*, to elaborate the conditions by which local actors were able to be agile, to adapt and to innovate in spite of the healthcare emergency and in coherence with their professional and personal values.

## 1. Introduction

During the COVID crisis, nearly every aspect of healthcare organization was affected, including research and development, human resource management, healthcare delivery, and conduct of clinical trials [1, 2]. A major worry during this time was to protect the continuity of care of chronic disease patients. One particular at-risk group was oncology patients, who face increased risk of severe complications of COVID due to a weakened immune system and because of their need for continued treatment to avoid disease progression and further complications [3, 4].

In France, cancer care was the most disrupted during the first COVID wave [5] which lasted roughly from March–June 2020, with activity returning relatively to normal in the months following. The medium and long-term impact on individuals, including survival, recurrence, and after-effects [6], in particular due to the disorganization of the circuit of care during the worst stages of the crisis, will however need to be studied in the coming years. At the current time, the severe lack of healthcare staff–in particular nurses and nursing assistants–is impacting the capabilities of the French healthcare system to completely recover from the crisis [7].

In France, a decision was made at the national level to prioritize continued treatment for cancer. In addition, some private non-profit cancer centers–as well as some specialized services such as pediatric oncology–did not manage COVID patients and could therefore function relatively as normal, even during the first wave, albeit with some human resource and protective material shortages [8]. Furthermore, to reduce the risk for vulnerable patients, it was recommended that some treatment protocols (in particular interventional radiology and chemotherapy) be adapted to ensure vulnerable patients spent less time at the hospital, or that triage protocols be put into place to ensure care for urgent patients. These decisions were in coherence with recommendations published by expert societies but adapted for the context of each individual hospital [9].

While all of these facilitating factors helped limit the impact of the crisis on cancer patients in France, they do not show the whole picture. The role of local actors–those working at the individual service or hospital level–and the adaptations and innovations they put in place also played an important part in helping healthcare institutions to be able to continue care for their patients. Our study’s ambition was to better understand what went *right* thanks to the capabilities of local actors and the changes they put into place that have had a durable impact on cancer care and research. Indeed, while the crisis severely disrupted them, it also provided an opportunity for action and adaptation [10].

## 2. Materials and methods

The research to be discussed here was part of a three themed research project funded by the Metropole de Lyon that investigated how oncology care was impacted during the crisis. Three teams researched the crisis from different angles: 1) patient perceptions; 2) impact on patient level quality of care indicators; 3) adaptations of hospitals during the crisis. The overall project was coordinated by the regional cancer network (Cancéropôle Lyon Auvergne Rhône-Alpes, CLARA).

The research to be discussed concerns the third theme, which focused on organizational adaptations and innovations during the crisis. It was developed by a three-person multidisciplinary and international team: a researcher in philosophy trained in social science methods, a biobank specialist, and a physician-scientist expert in translational research (the project lead). The methodology consisted of semi-structured interviews of 22 healthcare and management professionals in both a university hospital (*Hospices Civils de Lyon-HCL*) and a non-profit private hospital (*Centre Léon Bérard-CLB*, a member of the French Unicancer network of comprehensive cancer centers) in the Metropole of Lyon. The interviews were conducted by a researcher experienced in social science methods, with an interview guide (S1 Data) developed based upon the methods described by [11] that was first tested with a key person involved in the crisis at CLB. The criteria for selection of participants were: 1) professionals working in an oncology care organization or in an oncology research organization at the time of the COVID crisis; 2) those who had decision-making authority in their service, department, or at the hospital level; 3) professionals based in the Lyon, France; 4) those working either at CLB or HCL. Participants were sourced both from direct contacts of management staff and by snowball methodology [12], principally consisting of colleagues of the same specialties who recommended contacting other professionals in other hospitals/sites. These multiple sources to identify participants guaranteed that the researchers had access both to relevant persons as well as a diversity of professionals and specialties in cancer care and research.

In order to have a global view of oncology care, participants came from different professions, services, and specialties. This included 2 hospital directors, 2 vice-directors, 13 heads of service, and 5 persons working in research. In addition, participants came from a variety of different specialties and functions, including department heads in: head and neck (n = 1), stomach (n = 1), gynecology (n = 1), hematology (n = 1), pediatrics (n = 2), pneumonology (n = 1), endoscopy (n = 1), neuro-oncology (n = 1), as well as generalists in occupational medicine (n = 2), pharmacists (n = 1), nursing (n = 1), executive management (n = 4), and research (n = 5). The study was supported by the CLB and HCL hospital directors, who partook in the study as well as recommended further contacts in their hospitals. Where possible, there was a first introduction by a key contact person (such as the hospital director or colleague) to guarantee access to management staff.

All of the participants were informed of the aim of the research by written explanation and given an invitation to take part in the study. The interviews lasted from 30 minutes to one hour. Participants agreed to the recording of the interviews. The qualitative interviews were conducted until saturation. The interviews were recorded and retranscribed directly by the researcher with permission of the participants. In the transcriptions, all data was anonymized to prevent identification, including changing the gender/names. The study was reviewed and approved by the ethical reflection group at CLB (S2 Data).

In the interviews, professionals were asked to describe how their service/hospital/research was impacted during the COVID crisis, from the beginning up to the present day (the questions used are provided as a separate file in the manuscript). They were then asked to describe the adaptations and innovations that they developed during the crisis and that continued to be used in the post-COVID phase, as well as their use of the guidelines published by expert societies. This article will only discuss the results related to adaptations and innovations. As the research was conducted during the “chronic” phase of the crisis (March 2021-December 2022), this gave professionals the opportunity to reflect on what adaptations and innovations they put into place that have lasted in time.

The researchers analyzed the results using thematic analysis based upon an iterative and reflexive methodology [13]. The researchers examined the transcriptions and a final set of themes and questions were derived from the data (S3 Data). We also presented preliminary aspects of the research at: 1) a national conference focusing on social science perspectives of the COVID crisis; 2) an internal meeting of a social science research group at one of the hospitals involved in the study; 3) regular project meetings with members of the overall project team as well as the funder to discuss preliminary results; 4) a national meeting on adaptations of the COVID crisis in oncology in different French regions. These presentations gave the research team the opportunity to discuss results with other researchers and to understand the complementarity of the results with the experiences of oncology care provision and research in other regions of France during the crisis.

## 3. Results

“*We were a little bit in a comfort zone*… *Covid is like sleeping on a nail board*. *It taught us to be vigilant and to be efficient and to react to it*.*” (Caroline*, *an endoscopist)*

This section will discuss the principal ways local actors adapted and innovated in their structures during the COVID crisis. While these innovations were designed for the first wave, they were used throughout the subsequent waves of the crisis and had either become part of the normal provision of cancer care and research by the end of the research period (December 2022) or could be reactivated if needed for a new sanitary crisis.

By organizational adaptation, the researchers refer to intentional decision making undertaken by organizational members, leading to observable actions that aim to reduce the distance between an organization and its economic and institutional environments [14]. While early scholarship has focused on how the institution/management shape organizations (a top-down approach), more recent scholarship has shown the more reciprocal relationship between field workers and their institutional hierarchies [15].

By innovation, we refer to the definition introduced by [16], who propose that it be understood as changes that are original in context. The innovations that we will discuss are not necessarily “new” in terms of developing a completely new product or service, but they were original in context, as they were new to services, hospitals, and networks and have had an impact on cancer care post-COVID.

The results will be divided into two categories, encompassing: 1) new processes and resources to facilitate disciplinary and interdisciplinary work; 2) harmonization and streamlining of patient journeys. A summary of the results is detailed in Table 1.

**Table 1:** Innovation and adaptation during the COVID crisis.

Theme	Results	Level	Impact
New processes and resources to facilitate disciplinary and interdisciplinary work	Knowledge sharing through epistemic communities	Local and national-level coordination	Shared decision-making in the face of uncertainty
	Interdisciplinary coordination among services	Hospital-wide and city-wide coordination	More effective use of human and other resources in a time of resource shortage
	Increased use of telemedicine and teleconference technologies	Patient-healthcare professional level (teleconsultation); hospital-wide and national coordination (teleconference)	Benefits for healthcare provider/patient in some temporalities; increased coordination among hospital sites/services and for continuation of care and research
	New creativities in the care plan	Patient-healthcare professional level	Benefits for the healthcare provider/patient; a person-centered approach put into practice
Harmonization and streamlining of patient journeys	Harmonization of treatments	Hospital service coordination	Greater equality in treatment
	Streamlining procedures	Hospital service coordination and hospital-wide	Greater efficiency; clearer pathways for the patient

### 3.1 New processes and resources to facilitate disciplinary and interdisciplinary work

In this section, we will show how new processes and resources were put into place during the COVID crisis that enabled hospitals to be agile. This permitted greater local, national or hospital-wide coordination and helped professionals take collective decisions in the face of uncertainty and changing rules.

#### 3.1.1 Knowledge sharing through epistemic communities

*Healthcare*. As professionals were facing an unprecedent healthcare crisis in which decisions and information was updated almost everyday, they faced considerable uncertainty in how to adapt hospital services. One sustainable innovation to come out of the crisis was the creation and/or consolidation of online epistemic communities. This term can be defined as networks of knowledge-based experts [17]. These communities were often organized in an informal, spontaneous manner (either within existing groups or new groups created at the initiative of one or several professionals). They were made possible via online networks, in particular blogs, WhatsApp messaging services and/or group emails. At the end of the research period (December 2022), these epistemic communities continued to be active and enabled professionals to seek advice from colleagues. They continue to be used in the post-COVID period to share advice/experience on other issues relevant to their specialties. These online epistemic communities were original in context, as they allowed a new means of interacting among professionals in ways in which there had been limited coordination before the crisis.

These epistemic communities have been important in particular for professionals who felt isolated, notably those who did not have another colleague in the same specialty onsite. For instance, “Mark,” an occupational physician, said that he was largely isolated at the start of the crisis as he was the only person with his specialty at the hospital. During the first wave, but also the more chronic phases of the crisis, occupational physicians were obliged to make many complex decisions on how to protect hospital staff all the while ensuring sufficient human resources to continue cancer care. Mark describes the uncertainties faced by him due to constantly changing rules and procedures. According to him,

“*We had to follow directives that were both vague and precise…so for me*, *it was a huge job because I had to look for regulatory texts that were constantly changing…*.*we were in a blur because the social security did not answer and attending physicians were overwhelmed…at the beginning we knew nothing at all and so we were all alone*, *all alone*, *all alone to manage this*. *And so there was a blog of occupational physicians*. *We really exchanged a lot…we supported each other*. *I still get these messages now…*.*whenever there’s a concern*, *you can ask your colleagues*. *We know that hospital occupational physicians in France supported each other like this*.*”*

For this professional, the changing and imprecise nature of national directives [6] made it difficult to implement the recommendations. Given the necessity to guard professional secrecy to prevent identification of employees, these professionals could not turn to upper management for help. It is therefore not surprising that he repeats we are “all alone, all alone, all alone” to manage these urgent and difficult decisions. The use of an online epistemic community with occupational physicians gave him the opportunity to share information with other professionals in the same situation, all the while respecting his duty to confidentiality. The blog continues to be used to help occupational therapists agree to common strategies.

Other blogs were created at a more local level within the city, such as between nursing professionals or to coordinate care among liberal nurses and general practitioners. Both groups still exist in the post-COVID era. “Chloé,” a nursing manager, describes her use of epistemic communities:

“*I remember creating a WhatsApp group connecting all the care directors around us*, *saying How do you do it*? *What is the technique that you found*? *And in fact*, *there was a lot of communication between caregivers*, *between care managers*, *between us saying but what do you have in place*? *And now you’re closing visits*? *What are you doing or how are you doing it*? *We found that it connected us with the city like never before*.*”*

These kinds of epistemic communities helped the hospital to face the most severe and chronic aspects of the crisis, to create a link between the hospital and the city, and to exchange between healthcare providers of the same specialty, promoting disciplinary and interdisciplinary exchange. Epistemic communities were also organized in later stages of the COVID crisis by the regional health agency level (ARS) to consolidate the link between the city and hospitals.

*Research*. Epistemic communities also helped cancer researchers, in particular those working on live tissue cultures who had little institutional guidance from their structures. The need stemmed from isolation, in particular for those researchers working in the hospital campus where other professionals were not necessarily aware of their specific concerns. These researchers described how they often had to act alone in the face of uncertainty. For instance, “Etienne,” a researcher working with live tissue cultures who was concerned about the risk to his staff says,

“*I contacted the hygiene committee (at the hospital)…they had difficulty understanding the notion of cell culture*, *and I checked with the virology reference laboratory and they confirmed to me that indeed*, *if you have tissue that contains virus and you put it in culture*, *the virus is also cultured*. *So there is a risk…in a lab*, *there are still a lot of students*, *a lot of people*, *we can’t control everyone*…*and that*, *to me*, *seemed too great a risk*. *But it’s funny*…*well*, *I found the health committee strange*, *they hardly understood*, *in fact*. *They didn’t measure the risk…in any case*, *internally*, *there were no specific things for us*…*so we went looking for them* (other colleagues).*”*

Given the lack of guidance from his structure, this researcher mobilized his epistemic community to find answers about how to continue research and minimize risk for his employees. Without this essential information sharing among equals, it would have been difficult for “Etienne” to make a decision about how to continue research during this time.

#### 3.1.2 Interdisciplinary and inter-service coordination

*Healthcare*. In all of the interviews conducted with healthcare providers and researchers (n = 22), new forms of interdisciplinarity, inter-service, and intra-hospital coordination were highlighted as a positive aspect to come from the crisis. According to “Marie,” a pharmacist who worked in interdisciplinarity with several departments, the crisis, *“made room*. *It broke down a lot of organizational barriers*.*”* Both professionals from public and private hospitals shared hospital resources, including human and material resources. They also responded to urgent needs such as vaccination by working (often for the first time) between departments and specialties and took on cases from other hospital sites. This coordination in particular worked well within the same hospital group, where “hub systems” were put into place. These hub systems helped services facing resource shortages to find beds and not to refuse patients in need. Interdisciplinary coordination also worked well at the national level in “small” specialties such as pediatric oncology which could more easily coordinate on a wider scale due to a small number of cases. Several examples of these new kinds of interdisciplinary work are still in place and their impact is summarized in Table 2.

**Table 2:** Examples of new interdisciplinary coordination during the crisis.

Need during the crisis	Type of professionals mobilized	Impact
Vaccination campaign for professionals and patients	Pharmacy; occupational physician; healthcare volunteers from other services to conduct vaccination campaigns	Fast and coordinated vaccination roll-out for healthcare professionals and vulnerable patients
Transferring patients from a hospital service overburdened due to COVID cases	Healthcare providers in certain sites/structures that did not take COVID patients	Patients could be received by other services in a large hospital group, enabling care not to be delayed
Research on COVID	Interdisciplinary research teams (clinical trials)	Joint work from different research teams who worked together for the first time

*COVID reflection cells*. Interdisciplinary coordination was also reinforced at the hospital level via COVID reflection cells, which helped to decide overall hospital policy and to collectively debate difficult cases. During the crisis, it was essential to have different professionals’ views and sources of information in these meetings. According to “Anna,” a hospital manager,

*“That’s what collective decisions are all about*, *feedback from the field*. *It’s really important for the caregivers*, *for the administrators*, *to know what they are facing*, *to allow them to adapt*. *It’s pretty amazing how much discussion is needed in these meetings because everyone has information from different sources*.”

These new forms of interdisciplinary coordination enabled hospital managers and heads of service to better coordinate decisions and regulations during the duration of the crisis, as well as to find practical solutions through collective reflection. At the end of the research period (December 2022), these interdisciplinary coordination meetings either continued to be active, or were put on hold, but it was believed possible to reactivate them quickly. According to professionals, this kind of interdisciplinary work and coordination permitted the hospital to have a certain “agility” during the crisis, a capacity to act together which had not been present before and will be easier to reactivate in future crises.

One of the facilitating factors was the roll-out and widespread adoption of teleconference technologies. According to several professionals interviewed, this technology has permitted better interdisciplinary coordination between large structures, in particular between different sites at the large public hospital. In this past, the fact that professionals were spread across several campuses made it difficult to favor collective decision making. However, with the introduction of new teleconference technologies, meetings can now be held more frequently, allowing managers across services to be more agile and to take decisions together as a team. This has impacted COVID reflection cells, but also regular multidisciplinary meetings. As elsewhere [18], most of these meetings either continue in online or hybrid formats post-COVID.

*Limitations of interdisciplinary coordination*. Interdisciplinary and inter-service coordination was more difficult to organize at the city and regional level, particularly between different types of hospital structures (private or public). As “Etienne,” a management professional describes,

*“The hospitals were all alone*, *so we decided for ourselves*. *I think this is the major lack during the crisis*. *Everyone organized by themselves*. *All the hospitals have some capabilities to answer to the crisis*. *But there is no coordination between all the healthcare organizations in the region*. *In my opinion*, *this is the most important thing to do in case of a large crisis like COVID*. *We have to organize differently…the good level is not the hospital*. *It is the region or the city*, *but not (just) the hospital*.”

As we can see from this quotation, although online epistemic communities made it possible to share information at the city and regional level, it was more difficult to share resources as hospitals had different functions/statutes. The interdisciplinary coordination we have described here is therefore principally centered at the hospital level.

*Research*. A new interdisciplinary work was also put into place during the crisis for research in clinical trials, in particular via large European studies on COVID which mobilized different structures and services at a hospital site. In comparison to research on live tissue cultures (where researchers suffered from isolation within their hospitals and had to seek wider epistemic communities), those working on clinical trials benefitted from increased interdisciplinary coordination within their structures. In the post-COVID era, this has helped research teams to structure regular research work and share human resources among teams. According to “Martin,” a Clinical Research Associate (CRA), during the crisis,

*“We realized that we did not have an exhaustive vision of all the research personnel present at (the hospital group) in order to redeploy these personnel correctly and efficiently in the services that needed them most…since then we have been working on structuring ourselves and getting to know better the agents who work in research…*.*COVID made us realize that we didn’t have this exhaustive view*, *especially of our research staff*.”

This restructuring led to a creation of a new HR policy in the public hospital to allow them to centralize information on their research personnel. According to “Martin,” this will enable them to avoid skills loss and (hopefully) to better anticipate future research needs.

#### 3.1.3 Telemedicine and teleconference technologies

*Healthcare*. Telemedicine was greatly expanded during the crisis. At the time of writing, in the Lyon Metropole, it continues to be widely used in oncology care, including by different categories of professionals (oncologists, pharmacists, nurses). Table 3 summarizes the perspectives of the healthcare providers and researchers interviewed about teleconsultation, its value for the healthcare provider and patient, as well as the temporalities/spaces where it was not recommended by the professionals interviewed.

**Table 3:** Use of teleconsultation in care and research.

Temporality/space	Value for the patient/healthcare provider	Temporalities/spaces where it was not recommended
Pharmacy	Renewal of medication; patients lack time or cannot come in person; when patients need an additional explanation about medication.	First prescription with a patient
Post-procedure (surgery)	Keep the link with the patient before the next visit; permits healthcare provider to explain the surgical procedure when patient is more ready to receive information (at home, post-surgery)	Unfavorable outcomes
Communication about diagnosis	Announcing “good news” such as cancer remission	Breaking bad news
Patient/professional in their own home	Reduction of stress/burn-out both for the healthcare provider and the patient due to time gained	First consultation; new treatment
Care for fragile patients	Vulnerable and/or elderly patients living far from hospital (who do not need necessarily to come to the hospital)	Patients with few/low internet capacities or connections
Territorial inequalities	Increased access to patients that live far away, in particular from national reference centers and to bring care access to those who are out of the country/region	For those who can travel to the hospital site, most patients/healthcare providers continued to prefer in-presence care
Teleconsultation to inform patients of clinical trials and get electronic consent for participation	Helped continue research in spite of sanitary restrictions	May be used for future sanitary crises, but not ideal in normal clinical research settings

While professionals interviewed differed in their perceptions of the benefits for patient care, the crisis helped test the possibilities and limits and has had lasting effects into how cancer care is organized. The difference between the pre-COVID era and the post-COVID era is flagrant. According to “Anna,” a hospital management professional,

*“Eight weeks before the first lockdown*, *we did 25 teleconsultations*. *Eight weeks after*, *we had done 5*,*584…it’s something that has continued…even today*, *about 20–25% of consultations have been teleconsultations*. *It is almost paradoxical what I am going to tell you*, *but we observed that many patients could reach us more easily on the national territory and even beyond*, *in Europe in particular…they have transformed our practice and since then it has become common practice*.”

Our results suggest specific temporalities in the care journey when telemedicine should be practiced by healthcare providers, as well as when telemedicine should not be used, such as breaking bad news. These results largely concur with other qualitative research conducted in oncology on the use of telemedicine in France during the crisis [19]. However, further studies are needed to understand patient perspectives and to better understand how they differ from healthcare providers. For instance, a qualitative study on acceptability of video visits for serious or bad news delivered from the patient perspective has suggested that there was no consensus on patient preference for video or in-person visits [20], a finding that differs greatly from the perspective of all of the healthcare providers interviewed in this research project which were categorically against it.

*Research*. During the crisis, and in particular during the first wave, teleconsultation was also used in research to facilitate clinical trials, whether it be for cancer research or for clinical trials for COVID research. Teleconsultations were organized as a first step by the doctor/researcher with patients and in a second step by CRAs to answer further questions and permit patients to give their electronic informed consent. This was permitted during the crisis because they had a special authorization from regulatory authorities to use electronic consent. This allowed clinical trials to proceed even during the most acute part of the crisis. Trial modifications with the introduction of digital health technologies and trial decentralization were also used in other countries during the pandemic [10, 21, 22]. While the participants interviewed believe that this was a “good possibility/alternative option,” they believed it is unlikely to be used on a wide scale in normal cancer research in France due to the reinstatement of regulatory restrictions. They also said that seeing the patient in-person was the best way to ensure their informed consent. International research [21, 23] suggests however that remote monitoring and e-consent for clinical trials will be envisaged for future trials, in particular as digital consent has been shown to increase participation among rural and minority populations [24].

#### 3.1.4 Creativities in the care plan

As in other sectors of healthcare during the crisis [25], this special period also led to new creativities in the care plan. These creativities were seen in cancer care in the Lyon metropole (as in the community in general) and this in spite of increased workloads. This took the form of experimentations of arts methodologies to enhance dialogues with patients, particularly among nurses and other paramedical professionals. For instance, according to “Chloé,” a nursing care manager,

*“I’ve never seen so many caregivers show their creativity*. *One started drawing and making comics for patients to explain their illness…we made comics*, *newspapers*, *there was one person that made drawings with the patients and exhibited the drawings*…*one started to make music and brought in instruments to give out to the patients…I’m thrilled to see all the movement and talent coming out of our teams*.”

These new creativities were important not only to the patient, who was able to experiment with art practices to find alternatively ways to express and understand their illness, but also to healthcare professionals, their quality of life at work and their sense of purpose in their professions. According to this professional, these creativities are part of an ongoing reflection on how to improve healthcare and will help work toward a more person-centered perspective in oncology in the years to come.

### 3.2 Harmonization and streamlining of patient journeys

Healthcare professionals also took the opportunity during the crisis to find ways to better harmonize treatments and streamline procedures in their services. While this was not a phenomenon across all those interviewed, the crisis was an opportunity for a few heads of service in both the private and public hospital to improve their organization. While harmonization and streamlining are related and may collectively increase efficiency in hospital organizations, we define harmonization as a consistent approach to treating similar cases (such as harmonizing cancer care organization in a specific service so that it is the same for everyone). By streamlining, we refer to changing processes to increase efficiency and reduce duplicative views [26].

#### 3.2.1 Harmonizing treatment decisions

The interviews showed that the level at which treatment harmonization was possible during the crisis was in an individual hospital service, where there was a capacity to act quickly and with other professionals to harmonize treatment protocols. While these adaptations were often initiated by the head of the service, they worked on the ground because they were agreed and reviewed by the healthcare team. In addition, while these changes were made *for* the health crisis, they also accelerated transformations in hospital functioning that have lasted in the services which experimented them. As “Marie” (a head and neck specialist) explains,

“W*e were not really affected (by the crisis)*. *An explanation is that we reviewed our processes…*.*we said we clearly need to* ***harmonize*** *the prescription of treatment among MDs*. *We know that MDs by definition…have to have the freedom to decide what they do for their patient*. *Here we were in a crisis and potentially a big and long crisis*. *So we needed to refine things…we need to say*, *sorry*, *no*, *let’s everybody do the same depending on the urgency*, *depending on the complexity of the pathology*. *So we divided the treatment into four categories…*.*these were reviewed every morning…we believe we did not put patients in danger*. *We strongly believe it…*.*we all agreed together…and everything was really smooth at the end*.”

As this example shows, harmonizing treatments among professionals in the service was done both from a perspective of working toward equality (treating all patients the same), but also with the ambition of improving organizational efficiency (to improve organizational processes in a time of resource shortage but also for the future). As Marie’s example shows, this adaptation put into tension the individual autonomy of clinicians (who generally have the freedom to decide what to do for the individual patient even if they generally follow recommendations discussed at the tumor board in line with standard-of-care guidelines) with public health needs. It is however telling that Marie, as head of service uses “we” in explaining the context of these processes of harmonization and puts an emphasis on collective agreement and review, without which support for this harmonization would not have been possible.

#### 3.2.2 Improved processes to better support patients

*Healthcare*. during the crisis, hospital services sought to reduce the duration of hospitalization, a process already underway that was accelerated during the crisis. In the face of this new reality, two heads of service adapted by initiating new or improved processes to better support patients in healthcare pathways. According to “Colette,” a gynecologist:

*“This (crisis) made us realize that there are many things that can be done more in an outpatient setting…with COVID*, *we tried to shorten stays as much as possible…it also forced us to anticipate and take care of things beforehand*. *The woman who is going to be hospitalized and who is going to go home in the evening*, *she cannot go to the pharmacy beforehand*. *So*, *we have intensified the prescription of medicines in advance*, *so that she can go to the pharmacy before the operation*, *so that when she goes home*, *she rests and she has what she needs*. *We also now call the patients back to see if they’re okay*…*we accompany them*.”

According to “Colette,” although these procedures were beginning to be put in place before the crisis (as she expressed it, they are part of the “evolution of medicine”), the crisis forced professionals to rethink their care plans to better support patients. These new processes gave a realistic means of allowing patients to be able to transition to home care on the same day of the surgery. While reducing hospital stays was a reality for many hospital services both leading up to and during the crisis, this is not to suggest that replacing inpatient hospital surveillance with a (relatively unsupervised) hospital outpatient setting is necessarily the best option, particularly in some procedures which may incur additional risk; however, the reality of the acceleration of these processes did enable a few professionals and services to adapt to better anticipate and accompany their patients.

*Pharmacy*. New processes were also put into place to support patients who normally received medication via the hospital. These processes were put into place in both the public and private hospital. Given the necessity to put into place teleconsultation during the crisis, this led to new care processes for patients. According to “Benjamin,” a pharmacist:

*“It allowed us to think about how we can transmit information to patients when we are remote*. *Because we don’t handle the medication box and so on to explain the intake plan…it also allowed us to measure the proportion of patients who could be receptive or well equipped for remote consultations…we (also) switched to remote consultations for patients who were receiving treatment in clinical trials*, *something we had never done before*.”

According to “Benjamin,” these adaptations and innovations are helping to rethink pharmacy care post-crisis, and in particular the appropriate use of teleconsultation to reduce the burden for the patient and ensure they had the resources for responsible use of their medication. This was also the case for research and follow-up of patients in clinical studies. Pre-COVID it was not possible to get the treatment in the city pharmacy and it had to pass through the hospital pharmacy, which resulted in considerable time burden for patients who did not come from the city. During the crisis, pharmacists set up networks to deliver treatments to patients via city pharmacists, ensuring continuity of clinical trials during this time and giving benefits to patients in terms of medication access.

#### 3.2.3 Streamlining healthcare pathways

Two oncologists (heads of their service) interviewed were able to use the COVID crisis to refine their procedures in order to optimize planning schedules in their department. This gave not only a clear plan of action for the patient, but also helped to better organize their services to become more efficient. For instance, according to “Marie” (a head and neck specialist),

*“We said we need to* ***streamline****…*.*the day the patient comes to the CT*, *he agrees*, *and he will have his first treatment two weeks later*. *It’s a different way of organizing it…it was for me really big plus in the time of COVID crisis and we still apply it today…in other words*, *by* ***streamlining*** *things*, *by really maximizing the slots that we booked for a particular patient*, *we minimize the losses and this was somehow macroscopically an advantage for department…for me it was a really big plus in the time of COVID…we still apply it today*.”

As we can see from Marie’s account, the value of efficiency is prioritized to enable the department to “minimize losses.” However, for “Marie”, this streamlining also represents a benefit for the patient, who knows concretely when they will be treated and therefore has a clearly defined care pathway. By streamlining these processes in her services, “Marie” says they may also ensure greater equality, as each patient now has the same treatment delay.

## 4. Discussion

Healthcare professionals and researchers interviewed were surprised that they were able to be “agile,” to change, to innovate in their hospitals, which had always been viewed as large bureaucratic machines. According to “Marc,” a management professional, *“usually we have the traditional picture of the hospital which is a big thing*, *very difficult to move*. *The COVID crisis shows that hospitals are much more reactive than we thought they were…we showed that we are able to adapt*, *to face very unusual situations*.*”* While the relaxation of certain rules and the prioritization of cancer care during this time were facilitating factors for innovation, this is not enough to explain the creative period that was experienced in some structures and services. In this discussion section, what will interest us is how local actors–healthcare professionals, management staff, and researchers–were *capable* during the healthcare crisis, enabling them adapt and innovate to ensure that patients did not lose out.

In this section, we will articulate the project results with the capabilities approach, an interdisciplinary approach developed in social sciences. The capabilities approach (CA) is a social justice approach that seeks to better understand the real opportunities individuals have to act. It was originally developed by the economist Amartya Sen and later conceptualized in philosophy by Martha Nussbaum. The approach has been used extensively in health, especially to develop new initiatives in health promotion [27], health indicators and interventions [28], and to think about care for minority populations [29], although to our knowledge it has not (yet) been used to understand the capabilities of local actors during the COVID crisis.

In this section, we will first of all give an overview of the approach and our use of it, before articulating the approach with the project results. First of all, in the CA, the level of analysis is what the individual person can actually *be* and *do* (their capabilities). Therefore, in the approach, we will (not) only be interested in what a person’s individual *capacities* are, but their real opportunities to exercise these capacities in their environment (their *capabilities*). An example in healthcare is in the doctor-patient relationship: for instance, patients may have adequate levels of health literacy to enable them to participate in shared decision-making; however, they need to be able to exercise these capacities during the consultation. In other words, the doctor must both be willing–but also have institutional support–to give patients the time and space for shared decision-making. This is the difference between individual *capacities*, and *capabilities*, which takes into consideration how persons can realistically act in their social environment.

An influential thinker in the CA is the philosopher Martha Nussbaum. Her ethical and political project seeks to guarantee that a certain number of capabilities are enshrined in each country’s constitution and to put an obligation on governments to ensure everyone has the opportunity to develop their capabilities. She argues that her list of capabilities are the minimum to guarantee a life with dignity [30, 31]. Her list comprises the capabilities of: 1) life; 2) bodily health; 3) bodily integrity; 4) senses, imagination, and thought; 5) emotion; 6) practical reason; 7) affiliation; 8) other species; 9) play; 10) control over one’s environment.

While Nussbaum meant this list to be holistic (i.e. we cannot live a life of dignity without developing all of these capabilities to a minimum level), both research and policy orientations may focus on certain capabilities which may be more relevant than others in certain contexts. Therefore, in order to articulate our project results, we have chosen to focus specifically on three of the capabilities from Nussbaum’s list. These three capabilities include 1) practical reason, 2) affiliation, and 3) control of one’s environment.

### 4.1 Practical reason

The first capability that will be important to our discussion is practical reason. This capability involves critical thinking about what life we want to achieve and how to get there. In an ethical perspective, the capability to search for and pursue our version of the good is central to our self-worth. It helps us to reflect on our personal and professional values and to plan our lives in coherence with them.

An important means of exercising our practical reason is a reflection on our personal and professional values. In healthcare, these values motivate workers in their relationships with colleagues and with patients and families and give them a sense of purpose in their professional lives. As the project results show, the crisis confronted local actors’ values (personalized care, equality, solicitude) with the realities of the sanitary crisis and led to what “Adam” (a hospital manager) described, as a *“scene of major questioning”* that has continued to this day. According to “Chloé,” a nursing manager, *“we have never questioned values so much*, *why we do the job*, *what are the values that we have sought together*.*”*

The questioning of values in turn facilitated durable changes that will impact cancer care (and other healthcare) in the decades to come. Among the project results, we can see that this questioning led to the development of arts methodologies; thinking about equality, which led to greater treatment harmonization; or the creation of new epistemic communities with peers and new forms of interdisciplinary work in recognition of the importance of collective reflection. While these values were present before, it took the shock of the sanitary crisis to bring them to center stage and to create the space to experiment new methods.

Therefore, even with (and despite) the healthcare emergency, the capability of practical reason was important for healthcare providers and researchers to be able to adapt their care and research in line with their values. However, this practical reason, this reflection about their values, was not developed alone: it needed collegial reflection. To be successful, the capability of affiliation was also essential during this time.

### 4.2 Affiliation

*“The most important thing if there is a crisis*, *is to try and resolve the problem all together*. *I am sure that to share the problem*, *we can find the solution all together*. *In the case of a crisis*, *there is no other way*.*”* (Etienne, a hospital manager)

In this second discussion, we will elaborate the importance of thinking together, or what the capabilities approach calls “affiliation.” Martha Nussbaum defines this capability as, *“being able to live with and toward others*, *to recognize and show concern for other humans*, *to engage in various forms of social interaction; to be able to imagine the situation of another*.*”* [30, p. 34] This capability is associated with both being recognized and recognizing others. While we have thus far discussed the importance of choosing our own lives (practical reason), the CA also recognizes that we need others to be able to live well.

What we can see from the project results is that the ability to improvise and solve problems creatively—the process known as “bricolage”–was made possible largely by affiliation. As philosopher Paul Ricoeur [32] has proposed, in decisions of uncertainty, we can have recourse to a “good advice cell,” which he defines as a community of equals where several points of view are in balance. These “good advice cells” took the form of COVID cells, interdisciplinary coordination meetings, and online epistemic communities. They were a necessary factor in helping institutions to adapt and innovate and to develop collective practical wisdom. Etienne, a management professional, echoing Ricoeur, describes it as, *“there was a lot of decisions to take*, *but all the people were in this together*. *And there is a lot of collective intelligence…we had a lot of discussions together and we took the decisions all together*.*”* Similarly, Chloé, a nursing manager expresses it, as, *“Covid changed something about the fact that we had the capacity to act together…at the beginning each person was in his little corner…but then*, *we got into line very quickly…we structured ourselves and we did it together*.*”*

While this affiliation, this capacity to work together, was a necessary factor in enabling professionals to be capable during the crisis, this is not to suggest that the COVID period was conflict-free, or that this period did not cause value conflicts. For instance, “Marie,” although claiming that changing procedures in her department helped to better “harmonize” care, this process took time to negotiate, in particular with clinicians who continued to advocate for different rules for individual cases. This “conflict of values” between the doctor’s autonomy and public health needs has also affected healthcare in the post-COVID phase. Its long-term impact will need to be studied to better understand the capability of local actors in healthcare transformations.

The fragility of this affiliation should also be emphasized. While in the interviews, interdisciplinary coordination was a strong point in enabling hospitals to adapt during the most acute moments of the crisis, there was an evident rupture that was described in all the interviews between the first wave of the crisis (when everyone “gave their all,” working weekends, making homemade masks, etc.) and the more chronic phases of the crisis, when local actors were facing fatigue and burn-out. Indeed, due to the crisis facing hospitals due to lack of staff at the current time, it is at its breaking point.

### 4.3 Control of one’s environment

In this final section, we will discuss the capability of control of one’s environment. This capability is closely tied to the other two we have discussed. Nussbaum defines this capability as, “*being able to work as a human being*, *exercising practical reason and entering into meaningful relationships of mutual recognition with other workers*” [30, p. 34]. In other words, it combines the two previous capabilities mentioned (practical reason, affiliation), with the possibility to have a certain among of control over one’s environment (a capacity to act).

As we can see from the interviews, a facilitating factor in innovation was autonomy to decide how to best adapt care for their individual services. This included innovating at the individual service level (for instance the use of arts methodologies in a hospital service) to experimenting new forms of coordination at the hospital or even city or national level (new epistemic communities and new forms of research collaboration). It also involved experimenting with new technologies, such as teleconsultation, to find new ways to work with individual patients.

However, as Nussbaum’s capability has recognized, control of one’s environment also requires meaningful relationships and recognition from other workers. In this case, what healthcare managers needed was a certain amount of support (and confidence) from their hierarchies. Indeed literature on institutional resilience has shown that in order to facilitate quick adaptation, a combination of decentralization, self-organization, and shared decision-making is needed [33]. Or as “Marie,” a pharmacist describes it,

*“The top management trusts us*. *And it’s the same thing*, *I am underneath (him) but underneath me*, *I have people*, *I have effectors*, *I have relationships that go up like that and then I have actions to carry out and work in collaboration with the people who are under me and again I entrust their actions*. (The management) *can’t decide on everything*, *they can’t be on the ground of everything…it goes up*, *down*, *up*, *down and we talk to each other on all the axes*. *This shows the agility of the organization and that’s why it’s successful*.”

Based on this capability, we can understand that innovation was facilitated thanks to: 1) the existence of a semi-decentralized hierarchy; and 2) recognition of each person’s specific expertise in the hospital structure. In the first case, this enabled local actors to have autonomy to make specific changes in their individual services. This helps explain the number of innovations but also the variation in innovations developed during the crisis, from changes in triage protocols to service-level decisions on streamlining and harmonization.

Secondly, recognition of each person’s expertise also played an important role in helping healthcare actors gain the capability to act. As we can see from Marie’s quotation above, “we talk to each other on all the axes.” To be capable of acting, even within a semi-decentralized hierarchy, there was a need of *mutual recognition* along the hierarchy, from the top management to their local service managers, from local service management to their staff. Furthermore, outside of the individual service, mutual recognition was necessary for COVID reflection cells, where all points of view were necessary to understand the situation and take a decision. In these interdisciplinary groups, each professional was in their role, with a specific expertise to bring that was recognized and valued. Finally, this mutual recognition was valued in online epistemic communities, where individuals leaned on each for advice and support.

In conclusion, these two factors, a semi-decentralized hierarchy and the ability to have mutual recognition from other colleagues, enabled local actors to have a certain amount of control of their environment. Along with the capabilities of practical reason and affiliation, this made them capable of innovation and creativity, in spite of (and thanks to) the sanitary crisis.

## 5. Limitations

This qualitative study was conducted in one French region (Metropole of Lyon). Therefore, it cannot attest to the situation in other French regions, although feedback on our research in national conferences suggest that experiences were similar elsewhere. In addition, as many of these adaptations and innovations discussed by professionals were practiced with professionals in other regions (*e*.*g*. online epistemic communities), this does give us confidence that they were similar elsewhere. In future studies, it is suggested: 1) to investigate how many of these themes corresponded with experiences across France, in other regions of Europe, and internationally; 2) to better understand how these changes have sustainably transformed healthcare and research practices.

Another limitation of our study is that we chose to study our topic with managers. There is however a need to better understand what paramedical professionals lived, in particular given the current healthcare crisis, which was described by most professionals as “a bigger crisis than COVID.” Further research on the impact and repercussions of the lived experiences of frontline workers merits further investigation and public health attention drawn to the issue to prepare for future health crises and ensure that these innovations are not lost. Furthermore, our study sought to interview senior staff rather than young (less experienced healthcare providers and researchers) who did not necessarily make management decisions, although they were partly recognized in collective decision-making processes. This group merits further research attention. Finally, it will also be necessary to integrate the patient perspectives of these innovations to understand how they will change their long-term cancer care [34].

## 6. Conclusions

Many of the innovations and adaptations discussed in this article were implemented within the cancer care continuum during the COVID-19 crisis and are relatively simple to put into practice: the creation of an online epistemic community to facilitate exchange, collective agreement on healthcare pathways for the patient, and new processes to better accompany patients in the transition to home care. However, they were made possible by three capabilities: practical reason, affiliation, and control of one’s environment. Healthcare institutions, regional authorities, and politicians need to help cultivate these capabilities in both their frontline and management staff to enable their healthcare institutions to be able to adapt, be creative, and continue to provide quality care in the post-COVID period, and especially to ensure that they can work in coherence with their values.

## Supporting information

S1 Data Interview guide. (DOCX)

S2 Data Ethical approval. (PDF)

S3 Data Thematic analysis. (DOCX)
