# Correction: What went right during the COVID crisis: The capabilities of local actors and lasting innovations in oncology care and research

**DOI:** 10.1371/journal.pgph.0003934

**Published:** 2024-10-31

**Authors:** Brenda Bogaert, Zisis Kozlakidis, Elodie Caboux, Julien Péron, Pierre Saintigny

The fifth author’s name is spelled incorrectly. The correct name is: Pierre Saintigny. The correct citation is: Bogaert B, Kozlakidis Z, Caboux E, Péron J, Saintigny P (2023) What went right during the COVID crisis: The capabilities of local actors and lasting innovations in oncology care and research. PLOS Glob Public Health 3(9): e0002366. https://doi.org/10.1371/journal.pgph.0002366.
