# Supplementary material for: What went right during the COVID crisis: The capabilities of local actors and lasting innovations in oncology care and research
Source: PLOS Glob Public Health. 2023 Sep 25;3(9):e0002366. doi: 10.1371/journal.pgph.0002366 (PMC10519589; doi:10.1371/journal.pgph.0002366)
Supplement: S1 Data — (DOCX) [file pgph.0002366.s001.docx]

**Interview Guide**

**Hospital/Research Organisation**

1. Please describe your role and responsibilities (including during the COVID crisis)
2. Can you tell me how your service/clinical research/hospital was impacted during:

a.) the first lockdown?

b.) during subsequent waves?

c.) at the current time?

1. What were the challenges during the crisis in your hospital/research organisation from your perspective that could be improved?
2. What adaptations/innovations have come out the crisis (multidisciplinary meetings, remote meetings, flexible working, etc?) Which ones do you continue to use?

**Awareness/use of guidelines during the crisis**

1. What guidelines are you aware of to organise treatment/research during the Covid crisis?
2. Where did you source these guidelines? (sent to your inbox, sent by hospital/research management, independent research, etc?)
3. What is your assessment of these guidelines in relation to the realities of the situation?
4. Please describe any situations in which the guidelines differed from national level policies and/or your organisation’s rules during the crisis?
5. For you, what was the difference among the guidelines produced during normal times and during the Covid crisis? Did you have more/less confidence in the guidelines produced during the Covid crisis? If so, why?
6. When looking for information on guidelines, what is the criteria for finding a good guidelines? (ie do you look at the authors, the organisation, your speciality, the level of evidence, etc?)
7. How do you integrate guidelines in your everyday work? (are they an obligation, an de-facto obligation, considered merely one opinion among others, etc?)
