# Supplementary material for: What went right during the COVID crisis: The capabilities of local actors and lasting innovations in oncology care and research
Source: PLOS Glob Public Health. 2023 Sep 25;3(9):e0002366. doi: 10.1371/journal.pgph.0002366 (PMC10519589; doi:10.1371/journal.pgph.0002366)
Supplement: S2 Data — (PDF) [file pgph.0002366.s002.pdf]

A l'attention de Mme BOGAERT et du Dr SAINTIGNY,

La cellule « Analyse des projets sur données » du Groupe de Réflexion Ethique du Centre Léon Bérard (GRET-CLB) a examiné en date du 17/02/2022 le projet :

ARCOVID

Qui lui a été soumis par Mme BOGAERT dans le cadre du projet global IMCOCA.

Résumé du projet

IMCOCA, (IMpact COvid CAncer) est un ensemble de projets de recherches coordonnées par le CLARA dont l'objectifs est de mettre en avant l'impact du Covid dans le cancer, à la fois du côté des patients et de leur entourage mais également du côté des soignants, cliniciens, chercheurs et de leur pratique. Les différents volets de ce projet sont menés par différentes équipes.
Le GRET a été sollicité concernant la partie ARCOVID (p.8 à 11 du document scientifique) qui a pour objet une recherche SHS auprès des cliniciens et chercheurs (essentiellement) concernant l'impact du Covid sur leurs pratiques et leur utilisation des "recommandations".

Remarques des membres du groupe:

Nous conseillons qu'une ***lettre d'information*** soit rédigée et remise aux personnes interrogées afin qu'elle puisse comprendre le projet dans sa globalité, son intérêt et son importance. Cependant, un accord écrit ne nous parait pas nécessaire, nous estimons qu'un ***accord oral dans ce contexte est suffisant***.

Le GRET émet un avis consultatif favorable à la mise en place de ce projet *sous réserve que les remarques émises ci-dessus aient été prises en compte.*

Lyon le 17 février 2022,
La cellule « Analyse des projets sur données » du GRET-CLB

Groupe de Réflexion Ethique
Centre Léon Bérard
28 rue Laennec - 69373 LYON Cedex 08
gret-clb@lyon.unicancer.fr

Membres de la cellule « Analyse des projets sur données » : C. B., J. D., S. G., F. G., H. M., O. P., D. P., S. T., E. V-C.
