# Supplementary material for: What went right during the COVID crisis: The capabilities of local actors and lasting innovations in oncology care and research
Source: PLOS Glob Public Health. 2023 Sep 25;3(9):e0002366. doi: 10.1371/journal.pgph.0002366 (PMC10519589; doi:10.1371/journal.pgph.0002366)
Supplement: S3 Data — (DOCX) [file pgph.0002366.s003.docx]

**Qualitative data set**

We have chosen to share our information as a qualitative data set, i.e. to share the relevant parts of the interview transcriptions according to the themes presented in the research. We have translated these retranscriptions from French-English. We chose not to share the full retranscriptions as it was impossible to fully anonymize the interviews (the population of healthcare managers and heads of service is located in one city/region. This means that if we share the full retranscriptions, the persons will be easily identifiable). Our consent process guaranteed that they would not be identifiable post-interview and in articles published. This is an important concern in qualitative research and was important to our study as it may prevent respondents from giving full/honest answers.^[[1]](#footnote-1)^

We also believe that this grouping will be more helpful to future researchers, both because the interviews were originally conducted in French but also because the grouping by themes can help researchers to further explore these themes in the future. Where possible, in addition to the themes, we have also included the overall descriptions on the situation to enable data sharing on the situation during the COVID crisis and the reorganizational choices that were made. Table 1 summarizes the research themes described in the article.

| **Theme** | **Results** | **Level** | **Impact** |
| --- | --- | --- | --- |
| New processes and resources to facilitate disciplinary and interdisciplinary work | Knowledge sharing through epistemic communities | Local and national-level coordination | Shared decision-making in the face of uncertainty |
|  | Interdisciplinary coordination among services | Hospital-wide and city-wide coordination | More effective use of human and other resources in a time of resource shortage |
|  | Increased use of telemedicine and teleconference technologies | Patient-healthcare professional level (teleconsultation); hospital-wide and national coordination (teleconference) | Benefits for healthcare provider/patient in some temporalities; increased coordination among hospital sites/services and for continuation of care and research |
|  | New creativities in the care plan | Patient-healthcare professional level | Benefits for the healthcare provider/patient; a person-centered approach put into practice |
| Harmonization and streamlining of patient journeys | Harmonization of treatments | Hospital service coordination | Greater equality in treatment |
|  | Streamlining procedures | Hospital service coordination and hospital-wide | Greater efficiency; clearer pathways for the patient |

1. **New processes and resources to facilitate disciplinary and interdisciplinary work**

**1a.) Knowledge sharing through epistemic communities**

| 1. *Full description of the COVID crisis by the occupational therapist at the private hospital who described the situation facing them and how the creation of the epistemic committee helped to organize their work (named as Mark in the article)* |
| --- |

“The first. The first lockdown. It was a decision, you know, by the President of the Republic who shut everything down on March 15 or I don't remember the exact date in 2020. And at that point, it was as if the life of the country had stopped. And even at the medical level, regulations were coming out telling us that such and such an employee had to be at home, and such and such a person with such and such a pathology had to be at home. **We had to comply with directives that were both vague and, well, precise but vague, because there were no limits. If you had such and such a treatment, do you stay at home or can you come to work? And there weren't any. And for us, patients and employees, they had no medical interlocutor. So for me, it was a huge job because I had to search. I had to find out what was going on with the regulations, which were constantly changing and not very precise. I had to find out which employees worked, should work, shouldn't work. Were they too many? Was his treatment causing too much immunodepression to leave him in contact with patients or potentially with colleagues who might be positive? Or should he, could he stay?** And we were in the dark, because the Social Security, the Social Security doctors, didn't answer, and the person at the CPM didn't answer. The attending physicians, too, often didn't respond or were overwhelmed. So for the employees, we were the recourse. For the HR department, because here too, we had a lot of work to do, because we had to research the various pathologies that the employees had here, immunosuppressed patients who had undergone treatments that led to immunodepression or depression. Did I leave it, or don't I know how to send it? If you have 60 milligrams of corticosteroids a day, do you have immunodepression? If you have ten, you don't have it. It's that kind of treatment. If you have a history, which was afterwards, later in the months that followed, the learned societies came out with documents that helped us by telling us that with such and such a treatment, such and such a pathology in rheumatology, you but this, this and this, it's immunosuppressive. So at that point, we could leave them in or not. And we've had clearer guidelines as to where we can leave people who are really at risk. Of course, not in the ICU, not in the ER, but could we put them in departments with less Covid, etc.? **They helped us, but at the beginning we had nothing at all, so we were all alone, all alone, all alone to manage this.**

We had very few directives, so we followed them. And then, at the beginning, you know, at the very beginning of the lockdown, a lot of people were in lockdown anyway, so...and then little by little, it was more difficult to lift the effect for some to come back to work. Some wanted to, and always surprisingly, it's always the same in medicine. Some who had, who really had more, who could have had more fragility, wanted to come back anyway. And then there were others who didn't have much, who were very happy at home. Being paid at home isn't so bad, that's all. And then what? Then what? Afterwards, we know. Documentation came out and we were able to help ourselves. **And there are also blogs by occupational physicians. And so, there are sites, blogs, of occupational physicians. And we really exchanged a lot, a lot with the infectious diseases doctors who spoke.** And the professor of occupational medicine who gave us advice on SAM publications. Say, with these things that came out, that told me, here, you have to do this, this and this. We relied a lot on that, all that, all that and then SAMs, it's the alert messages, the ARS alert messages that send this or that to the establishments. MAS, that's what we relied on. And then, really, the occupational physician's blog**. We know that hospital occupational physicians in France have supported each other like this.** It was created by the professor. Anyone can get involved, and it's really been a great help to all hospital workers, especially because...**it's a really collegial discussion**, but really really. It's not at all the professors who give advice because they don't know the field at all in practice that's it, but they can give an occasional opinion. **Then it's the colleagues together.** I found this documentation, but that's how I do it at home. I work with infectiology at my university hospital, and he told me to do it that way. Ah, that's not the way we do it, listen...it was...didn't I tell you about the vaccinations? We had to get the vaccinations, but we had to get them in and give the documents. And that didn't work at all well either. At one point, it was horrible, horrible. so it's also in the application all anti-covid....there was a cinema...there were a lot of malfunctions, but there were certainly things that worked. But there were a lot of dysfunctions.

[Interviewer, later on in the interview: ] In normal times, do you use these blogs?

Ah yes, since the beginning. Since it was created, it was. It was created mainly a tiny bit before, I think. But really, I was able to press it. I asked questions too. I can give you some answers by saying, well, that's how we do it here. **No, no, no, huge. Not every day, every day, every day. But I still get them now. But at least now, people are on vacation. But as soon as there's a problem, you can ask your colleagues like that.** **It's really been a help. It's really been a great help. General practitioners, I don't know if they have it, I don't know, but it's more for times of crisis, you could say, because for everyday life, we don't feel the need as much.**

| Full description from another occupational therapist (at the public hospital) on the situation in the hospitals and epistemic communities built by occupational physicians |
| --- |

**“**It's been completely reorganized, so to speak. Not to say unorganized, but reorganized. Let's just say that I think we all pulled together pretty well. Well, they told us, well, there was the speech of March 15 in the speech of the President of the Republic, state of war. Personally, I was a bit worried, thinking that we didn't know this pathology, we didn't know how to treat it. At the beginning, this was the case even among us, in the occupational health team, but not of deaths, we were worried for the professionals too. Certain pathologies had to be avoided**. So we were, let's say, very anxious and stressed, but also very mobilized. And I think we all pulled together in the department. It was a huge workload for everyone, for the doctors, but also for the nurses, for the secretaries, really for everyone.** And that was it. And then, with instructions arriving and changing every day. So there was a big, big mental load. In the morning, you had to do things one way, and in the evening, things had already changed. In the beginning, we didn't know the pathology, but we got to know it, and so we did. In the beginning, we were, we had a lot of calls. So we had two call centers for calls from sick carers who had to be referred for screening or not. Knowing that at the beginning they had very few reactives. So I think that now, people who would have been screened three or four times, well, we were obliged to say no because they hadn't been in contact with a Covid case, because they didn't have a fever, and so on. There were flowcharts, but they were evolving. Then we called and were contacted by patients with pathologies that exposed them to severe forms of the disease. And that's when we saw the texts of French society. From occupational medicine. We also had texts from the French health authority (Haute Autorité de Santé), which were published on a regular basis. **And this famous network led by Professor JPG...and there were lots of discussions with occupational physicians, so it gave us a chance to ask questions. And we often had more or less the same questions, so what did we see? We were lucky enough not to be an isolated occupational physician, so we could ask each other questions. So there you go, but we had consultation lists. On top of that, we were asked to stop all our face-to-face visits, so practically everything was done by telephone. So another way of working from a computer point of view, we all got up to speed very quickly. Etc. In terms of videoconferencing too, it was quite unusual for us. We had regular Skype meetings, sometimes several times a day. So that was another big novelty.”**

| **Full description of the sanitary crisis and the importance of epistemic communities by the nursing manager (pseudonym is identified as Chloé in the article):** |
| --- |

“First of all, we saw a situation coming that didn't belong to us, because we saw other countries in trouble and with a feeling that we were very protected and even that we sent masks, that we had lots of people asking us. China needs masks, etc. and we were like yeah, we don't need...no problem. And so it's the feeling that we're protected and that we started this moment when we were, first of all, seeing the questions coming from SAMU and that it was meetings every week, with people looking at what was happening, the epidemiology. All of a sudden, we realized that there was a kind of difference between the outside culture, as soon as we left the establishment, and the inside, because we had begun to understand that we were faced with a situation that we hadn't mastered, and without knowing what it really was, without knowing...sincerely. I remember writing a diary because I was, I didn't understand at all what was happening to us. I had this feeling of starting to say, but is there knowledge? That's if there's knowledge, because in fact that's what it was, because the baselines didn't actually exist. And the first step, I'm having trouble getting my mind around it. It was. The only thing we were waiting for was for people to be put into lockdown, because we could see the curve rising. And as the weekends went by, the number of contaminated people increased and we had less capacity. Our problem wasn't necessarily that our patients were contaminated, it was that we had no more space in the ICU and we couldn't operate on patients with the risk that they would no longer be able to. So we started looking at our operations and saying, these patients really need to be operated on, and if they don't have an ICU, what are we going to do? And it was improbable to have that. And I did. **I remember creating a WhatsApp group connecting all the care directors around us, saying How do you do it? What technique do you find? I don't know. And in fact, we communicated a lot among caregivers, among care managers, among ourselves, saying, "What have you put in place? And now you're closing and closing visits? What do you do or how do you do it? You've got masks, you've got no masks, you've got gowns like we do in the rooms, and we had no idea what to do. There really was this feeling. We were waiting for the learned societies to talk to us, and we weren't really getting any feedback. So here, I started debriefing. Everything was briefer, debriefer. That is to say...I had one at 9 a.m. to 12 p.m. and one at 4 p.m. and zoomed in.**

First of all, it didn't have a zoom because there was absolutely no such thing. So I had an octopus phone, a kind, and I called all my cards because we understood that we couldn't all be in the same place because we didn't have masks. But whatever happened, we couldn't all be in the same place. So, and I felt like Charles de Gaulle in England saying, so I heard you do this this this and that. And in fact, there was no feedback because I felt I was just giving information. I had set up a sort of Excel spreadsheet online so that he could ask me all the questions before 9 am and before 12 pm, before 5 pm. And so I would take all the questions he had asked online and answer them point by point, because as I have around 50 managers, I didn't know how I was going to provide information or answer all the questions live. **So we set up a frequently asked questions section, where they could ask questions. If I didn't have the answers, I'd go and look for them, and so for three months we did this, three times a day from Saturday to Sunday inclusive.** We gave it a try, and then we realized that it was early. I said it was going to be a long-distance race, so everyone had to take a rest, because in fact, at the beginning, we weren't, we were taking more rest because we didn't know how to leave our teams with the uncertainty, and I understood that we were going to be in it for the long haul. I don't know how I realized that, because China was starting to tell us that it was happening again, that it had passed and was happening again. So we're actually, it's going to ease off, it's starting again. So we can't be happy that it's over, because it's going to be a long-term thing. And then, first of all, it was complicated because I really have it. I don't think I was sleeping and that's a problem. In fact, I saw a shrink here, who had put in a crisis management psychiatrist. I told him I felt like I was taking people into battle without weapons. There were no masks, no tricks. And I tell them go ahead, yes, there are Covid patients. There were a lot of Covid patients and I remember telling them they didn't need masks because we didn't know...one weekend we calculated the number of masks needed to change every 4 hours and the stock with the pharmacist. And I said, but I don't have more than 24 hours' worth of masks, so who do I give masks to? If I put that rule in place, why shouldn't others have it? And it was equity of equity, or equal treatment. So we preferred to make decisions. We don't have enough masks, so we don't give masks to anyone. And when we do manage to get masks, and fortunately, I don't know how long we didn't have masks. We were delivered afterwards by the army, we were delivered by the army, all the establishments had a delivery, but we didn't have many. We had a few, so afterwards we told who had the right. I remember talking about the mask circuit, then the gowns. As we were going to wash them, we bought washing machines to wash the gowns. I contacted the seamstresses to design the gowns and the garbage cans. There were no specific garbage cans. The circuit like that, it commandeered everything and so I felt like I was fighting. And at one point, I remember very well. I think I was exhausted. I took a sheet of paper. I went like this. I said I took it, I drew a sword and I walked into the medical director's office. I said I'm giving up, I can't take it anymore, but actually I can't take it anymore. I don't know. I can't fight anymore because I don't have the strength. I can, I'm exhausted.

(later in the interview)

**Interviewee:** You mentioned the WhatsApp group?

**Nursing manager: There were two WhatsApp groups the WhatsApp group with private nurses, GPs, the town. In fact, it was the president of the URPS who organized it. And it still exists. And then there was the WhatsApp group, made up of healthcare managers, directors of care, from the establishments. And that made it possible. What does Covid stand for? We have two groups. Covid care.** There are no more masks, the virus is spreading slowly, no one is talking about it anymore. Between the war in Ukraine and monkey pox, everyone's tuned out. That was this morning at 7 a.m. Yes, the numbers are dropping, but hey. And what do we do? And who's going to get vaccinated? The question is, what are we doing? And it's risky because there are so many publications. Not really a place to be found. **And I found that it connected us with the city like never before. And then, it also gave me a quick Covid exchange, so here's the director of M. site, if you've heard about the special dispensation for students who could replace nurses. Etc. Thanks for the info. What did she ask? What did she ask? I'm looking for the e-mail address of someone who can find solutions at HCL. Was there anyone there? So we can finally talk a bit while I don't know everything at first we said what are we going to do? Hello, what are we going to do with the students because we had interns? We didn't know if we could keep the trainees, orderlies, nurses. Who can tell us what's going on with the schools? And the director said yes, because we're in them too, the nursing school directors. Training is being maintained. The distance for nurses has continued to operate. Training has not been interrupted. Only the i-ibot training courses have been suspended, as have the operating room nurses. It was interesting because, in fact, we're closer to city medicine.** It's hell for general practitioners. So we had that feeling too. **We weren't alone, were we? It's not that this moment of solitude was so strong that it's still there, nobody's left. Phew!** I didn't see any response from (inaudible), but now we're in competition. And that's the problem, now that we have so many more carers, it's the same CV that goes from one thing to another and it's distracting because we're a bit good. It's a race to see who can get the most caregivers for themselves. Normally, I'd organize an aperitif before the summer, because that's what I do very often. And now, I feel that if I suggest an aperitif, they'll say, "Wait, it's too much trouble. And then they're probably angry with me because I've taken some of their carers home, maybe because I'm angry with them, because they've succeeded in their mission with me. But in fact, maybe we don't resent each other at all. I don't think we resent each other at all, but in fact, there's a kind of misunderstanding that means we're perhaps in a bit of competition. I don't know about that. Personally, I feel there's competition between healthcare establishments. Then we're all exhausted. And at the same time, it's now or never to say let's overhaul the healthcare system (laughs). That's what drives me. It also allows us to say OK. Maybe the system wasn't good enough. **We're fixing it, we could have worked better with the City would work better. We'd like the liberal players like us to respond to the health needs of the region, much more than the hospital focused on how we should manage Covid and the whole region. We can't do it any other way. We have to centralize everything at the hospital.”**

**1b.) Research**

| Full quote from Etienne, a researcher, about the use of epistemic communities in research and a full description of what happened in research in the early stages of the COVID crisis: |
| --- |

“The main impact for my activity was to say, as patients were not systematically tested for Covid, so nobody tell me that I have to take, to analyze the risk, but I thought it was my responsibility, I know that I have staff that was everyday touching samples, it's their job. So they know how to protect themselves. And so okay, but I also...it was my responsibility also, I thought if I give to researcher, for me it was a risk...so at one point, so I, in fact nobody was worried about us, about the activity, but so I did some research, on what I could see in the literature to know the risk or not that I could make my staff and researchers take, knowing that the staff...we still have the knowledge of the risk and we have equipment, etc. So I quickly saw what equipment was needed. But the researchers - and especially for fresh tumors, most of them use cell culture, which is used in areas where there's a lot of traffic, and students, and I don't have the control, and I'm still responsible. **I checked with the virology reference laboratory and they confirmed that if you have tissue containing virus and you put it in culture, the virus is also cultured. So if you amplify it, there's a risk. So I couldn't just say to them, get some equipment, FFP2 mask, gown, etc. because then, I mean, we know it's aerosolized, so it can spread...as these are labs and there are lots of students, lots of people, we can't control everyone...and that seemed too great a risk to me.** **But it's funny...well, I found the hygiene committee a bit strange. They didn't measure the risk, I think, the risk in fact.** So I confirmed, I continued, I said "no" and so there are tissues that I no longer give to research, unless we manage to do tests on the patient 48 hours before and so for me everything that is at risk is open to the outside so ENT, lung, intestinal, intestinal tract ... and so we stopped and there were some researchers who were not necessarily happy, but yes ... so I thought it was not ...so for some...as there are some researchers who are also doctors, for the intestinal part, they've managed to get people to agree to do tests, to ask patients to do tests beforehand, afterwards it's typically borderline, because given that it's not standard practice in this hospital, in fact we're making patients do something that wasn't planned in their management, so it becomes a clinical trial where specific consent is really needed. In any case, it's been...well, it's been so common to do tests up until now that it hasn't been too much of a problem, but it's a bit of a limitation too, in the end. In short, for the intestinal, there was a doctor who set it up, so it was fine...for the lung, it's the only pathology where it was systematically skipped, and for the ENT I said no, so we stopped, until they released the complement in March, in France, so we said actually the risks are less for people, since we have to be vaccinated, so there you go. I said to myself, there's no point in doing that either...since staff are vaccinated, I thought, well, there's a risk, but it's maybe less than it was a year or two ago, when it wasn't. But we're in places where everyone has to be vaccinated, otherwise there's no risk. But we're in places where everyone has to be vaccinated, otherwise.

Researcher: so the risk was really just for the staff?

ST: well yes, especially for the researchers, and yeah, and especially for the researchers' environment, i.e. they themselves will be able to take equipment, but we're in places where there's so much passage of different populations that I found it difficult to control….and so when **I contacted my management again**, I said, okay, I'll reopen unless you tell me otherwise. And they said, yes, yes. Maybe I'm too surprised, but I'm at a level that means I can make this kind of decision, so there you go. I think I did the right thing anyway.

Research: I'm still surprised there wasn't a discussion at the hospital level?

**ST: In fact, it's because they don't have that vision of research. And yet some doctors are also researchers, but they see patients all the time, and they have the impression that we're worrying about nothing. And the notion of culture may still be a little abstract for some people, so yes, I too was surprised…**I had to insist several times, not too much on the fact that there was perhaps a risk, they said, ok if she wanted, but on the other hand I insisted a lot on the fact, not to stop the research or set up automatic tests, and that... no. With the exception of a few cases, some doctors or some people who played the game personally as doctors...

Later in the interview:

“I based myself on the recommendations, in fact. So it's not my management (laughs) who have...those that were made were often intended for doctors, for carers, so in the end it was me who set up...after the biobank on their side too, the anatomo-pathology, they also set up things on their side, precisely because they receive surgical parts and in addition they work...there's a first stage which is in macroscopy, so they're not "swat" at all and they're less protected than us…**and in any case, internally, there weren't any specific things for us...so we went looking for them, then we found information on the site...there's the French Microbiology Society which gave information and then there was also the US side, I know there was a site that talked quite a bit about sample handling, so there you go, that's how I did it.**

**The use of fresh culture, culture, I didn't see anywhere, so I went looking for information, and so I knew someone, and so, at the end of the day, it was a colleague from my class who's at the national reference center, who shared the recommendations sometimes, so I went looking for expertise when I didn't have precise expertise. In any case, I tried every time to find the answer to the question, so every time I didn't have a general recommendation, fortunately I was able to get in touch with someone, otherwise I'd have been in trouble (laughs)…”**

1. **Interdisciplinary and inter-service coordination**

**Table 2: Examples of new interdisciplinary coordination during the crisis**

| **Need during the crisis** | **Type of professionals mobilized** | **Impact** |
| --- | --- | --- |
| Vaccination campaign for professionals and patients | Pharmacy; occupational physician; healthcare volunteers from other services to conduct vaccination campaigns | Fast and coordinated vaccination roll-out for healthcare professionals and vulnerable patients |
| Transferring patients from a hospital service overburdened due to COVID cases | Healthcare providers in certain sites/structures that did not take COVID patients | Patients could be received by other services in a large hospital group, enabling care not to be delayed |
| Research on COVID | Interdisciplinary research teams | Joint work from different research teams who worked together for the first time |

1. **General quotes on interdisciplinary coordination during the crisis**

| Description of the role of interdisciplinary work in the private hospital (pseudonym is identified as Chloé in the article): |
| --- |

“Did the Covid change anything? In any case, it changed something in terms of **our ability to act together. Doctors and nurses, logisticians, it brought us closer togeth**er. But at the same time, it also showed the emotional elevator that the carers had. But I feel useful because I'm helping to make the world a better place, because everyone's looking at us in a different way. But then we had to communicate with the teams about the vaccine, and we got...I felt I wasn't strong enough, as strong as the social networks. There was a lot of misinformation coming from the social networks and the caregivers were very suspicious of the vaccination.”

| Description of the role of interdisciplinary work in the public hospital (neuro-oncology) |
| --- |

“We're lucky at the public hospital, where the management really did a great, great job of communicating. The doctors were actually very supportive of the first wave...which was a good thing, because there were so many of them. As all the medical practices were closed, the former heads of clinics who had stayed in town came to help us. Young retirees came back. Nurses, orderlies, medical students and surgeons who were no longer working started phoning families to keep them informed. It was great. Quite good. The wartime atmosphere was not found in the waves, one, two, three, four, five, six. So afterwards, it was a period of discouragement.”

| Description from occupational therapist about interdisciplinary coordination |
| --- |

**“In any case, for me, there was more mutual aid, much more mutual aid, it seems to me. But yes, there was mutual aid….between the different people at the (hospital), it seems to me.** In any case, at the medical level, I felt it. I teased the pharmacist non-stop (laughs). He was very patient despite his workload. We stuck together. I think he really pulled together during the crisis…but at (the hospital), there was a lot, there was a lot of mutual aid. People don't count their time. Apart from a few...yes...there have been a few people who have hidden away when they could. But on the whole, all the people were really devoted to the establishment, to the patients in the end, to keep things running. Personally, I really feel that people didn't count their hours in a crisis situation. Most people.”

| Quote from vice director of private hospital on collective intelligence (but also its limits in terms of fatigue with time during the crisis) |
| --- |

“That's exactly the point. Because during the first wave, in fact, it was easy. Easy, because all the strength of the hospitals are directed on the COVID. And, for me, it's, it's time consuming, but it's relatively easy to organize the hospitals, because we stop some activities. And then we focus just on the COVID. **Okay. So, there is a lot of decision to take, but all the people were in this all together. And there is a lot of collective intelligence. And, in fact, it's easy. And it's a great moment, in fact, because the result of collective intelligence and it's not so often after that, it's more complicated because all the people want to return to normal activities.** And all the people want to to care, to care the patient to make surgery or radiotherapy, etc, etc. And to make the palliative care for example, or the third line of chemo and cetera. That's why it's more difficult. Then we have also another point that this is the fatigue with time, all the people, especially the nurses, and all the paramedics are a little bit tired with the first wave. And it's difficult to restart. We have the same problem today. Because it's very difficult to recruit or to recruit the nurse. Not for the medics but for the paramedics. Well, for that, a huge problem for the recruitment. It's very difficult to we have a lot of people who leave her job. To make something else: this is a life project. That's a little bit different. And for the moment, it's difficult. Before the COVID, each week, we have some, we received some curriculum vitae for nurse etc. So we have just to choose on this this CV, the new nurse or the new paramedics. Now we have no CV. We are obliged to search the new professional for the hospital. That's, that's different than before. For us. This is the I think the most important thing after the COVID this is the motivation, the sense of purpose.”

(later in the interview)

In the normal time, all the groups work side by side. And then with the COVID, there is more transversality. Perhaps this is the difference. The other word is the use of video conference, but it's not a new thing. It's not so bad. This is not probably the unique way for meeting but it allows...it's small meetings because a lot between a lot of people it's easier for small meetings between people who know other people we all know each other and that's a good way to have a quick meeting. It's a good thing. I think we keep this kind of meeting, I think.

The most important thing is if there is a crisis, is to try to resolve the problem all together. I am sure that to share the problem and to find the solution all together. There is, in case of crisis, there is no other way but sometimes if all the people do not agree all together, you have to decide and to slice the cake...but I think we will use the same method (in the case of a future crisis).

1. **Vaccination campaign**

| Full citation from pharmacist about the role of the pharmacy during the COVID crisis and the role of interdisciplinary work |
| --- |

“For the first wave. In fact, what happened was that, of course, we were totally unprepared. Even if we were expecting something, we were still expecting something. It's as if you know there's a wave coming, but you don't know what it's going to be like. If it's going to be strong, it's going to be long. If you don't know, you don't know what to do. We knew that our Italian colleagues, because they had it a fortnight before us, and in particular the cancer center in Milan, which was having enormous problems because it no longer operated here, had simply become a center, a center for COVID patients. So he'd stopped chemotherapy altogether, and was sorting patients out like in the war. In other words? In fact, it was treat, don't treat, if he's too old, but don't take into account... so it was war medicine and so the only thing we had before and after. And then it happened. So there's the context, let's say of the President of the Republic who is, who explains everything, all countries stop, etc..And we continue to work, not normally, but even though we weren't a level-one center, but rather a level-three center. Because we weren't the ones on the front line. In quotes. In fact, we've been hit hard. **In terms of activity, that is. It makes way, it breaks down a lot of organizational barriers.** With the white plan now well underway. In the white plan, what was again very good was that there were all the trades and there are a number of levels of management. In other words, yes, there was top management. But there was also the stretcher-bearer manager. There was. And the good thing was that this was a posteriori. **But the good thing was that everyone had a right to discuss, and it's not a decision-maker. That's the best part. No, it was really collegial.** On the other hand, it was very, very heavy in the sense that it involved a lot more work, that's for sure. And physically too. Once again, even if we weren't at level one, it was very, very, very stimulating. It was very interesting, but we paid for it a bit later...so pay, pay, you had to take vacations as it was in that sense. B**ut it was really very, very stimulating because it was all new. We got together on Saturdays and Sundays, etc.** So afterwards, in the story, we had to cope with things like what's happening in Ukraine, things that are ten times worse because there are deaths, ten times more deaths here in the context of this wave of the virus. But in concrete terms, we've had to deal with shortages. And then, just like when you go to war, I suppose you immediately lose 25% of your forces. In other words, there are 25 percent, 35 percent of the staff who are no longer there, either for the right reasons, because they have a sick child, or for the wrong reasons. Clearly, we have to mention them anyway, because there are some cowards out there. And I say it like that, there's no problem in saying it. In other words, there are people who invent a hypertension, who won't come. And that's part of the 25%. So we immediately lose 25% of the strength with more work. So at least the work is different. And that's it. After that, all our external correspondents are also having difficulties because there are no more resources, because the country is blocked. But it's also because there are more resources worldwide. And so, from what you've heard, we have a shortage of hydroalcoholic gel. That's how it started. A shortage of masks is not something the pharmacy has to deal with. But since there have been shortcomings, people have been managing this kind of thing. **In fact, I'm the one who took over. So we took over and that means we have to find our own way**. Once again, thanks to the white plan, we say we have these problems. Then everyone tries to find an industrialist who makes, who makes alcohol. And then, at the same time, he does something else. And that's how we manage to get deliveries. And then, because we're on our own, we manage to get hydroalcoholic gel. Then, once again, there's the question of safety, which isn't my job. So that means getting in touch with these people, checking what it is, having it delivered. Once it's delivered, I'm not the one who manages it. That's exactly what's happening with oil at the moment, in France, where people, for stupid reasons, aren't going to take a liter of oil every four weeks, but they're going to take six, and all at the same time. So we're bound to run out of hydroalcoholic gel, and it's the same thing, even if we had plenty of the kind at any price. And then in ten seconds no, that meant we had to ration like we did with narcotics. So in fact we set up. I'm setting up an organization, which isn't always in the same place, because we've had thefts at one time. Masks have been stolen during research. We lost 25,000 masks. Something like that right from the start, people have been stealing masks. So hydroalcoholic gel was the same thing. So that means it was contaminated. It was located in a secure place, in the pharmacy, under alarm. Next came the masks. Then, almost at the same time, we put on the masks. So here we go again. Then we had an industrialist pass us some. And then we succeeded. In the end, we never ran out. But as we were almost ready to run out, I bought some fabric masks…but they were big rolls of fabric. So again, what do you do with that? So it was up to me. Again, it's not my job. And so that means that, on the other hand, in the plan, in the organization of the white plan, I ask if there are people who have staff, who do nothing and who aren't even here because they're not nurses. And so, in fact, I set up a dressmaking workshop with these people. I got some people from my unit to tell me that the operating theatre had been shut down. I said I was in charge of sterilization. So I had four or five people from my unit come back, plus some people from the ISD. And so I requisitioned the works council, the premises of the works council (the establishment's social committee). So we had people coming in with scissors, irons etc., and so we made 10,000 masks at the same time, washed them and bought a washing machine. In the end, we managed to get through that phase. Then there was the problem of vaccines, so vaccines, then vaccines. It was complicated because we didn't know what to order. We were, we had all the capacities here to be able to store at -80 because we have -80 refrigerators, it's all monitored. But the State didn't want...It wanted a single pivotal establishment, the hospices, to deliver, and it wasn't me at all. It took me two or three hours a day to find out how much to order. So there you have it. Then we made the decision. Well, I made the decision to produce the syringes in-house, in the pharmacy and not in the departments. Why was that? Because even to lose, to avoid losing as few doses as possible. So we didn't lose any doses**. So we vaccinated all staff and a large number of patients. Because we set up a vaccination center. In the space of a week, we set up a vaccination center. So that's it. And now it's normalized, we're doing more vaccinations.** At last, things have returned to normal. There's nothing special. We do antibodies, but antibodies aren't very complicated. There's very, very little preparation.

Later in the interview :

«  That's what I said at the beginning. **That is to say, in the white plan meeting, in fact, we took turns expressing ourselves, or each person who needed to say something, to express themselves.** And if there was a problem afterwards, there was the problem of masks. OK, so I said OK, what I propose is. I'll take care of them, I'll do this, this, this. And then afterwards, the director says OK, the others talk, etc., you do it, you do it and I organize it. And then I say here's how I want to organize something, it's OK, it's OK. And then, never mind, the barriers, even if it's not me who says who's usually busy, it's me who takes care of them. And then I set up an organization and it's always validated. In one case, it was also interesting, that in the organization, as it is in fact, I like the term war because I think it's not bad. And in war, the enemy is variable, he changes and the virus is the same, it was variable, the people the virus was, the shortcomings were variable, the equipment etc. and that means you have to adapt when? When there was no longer a shortage of hydroalcoholic gel. But in fact I'm back to normal. I gave it back to the person who was taking care of it. He took care of it again. And it's the same with the masks and the overalls. **It's in times of crisis. Well, we adapt, we make a different organization. And then, as soon as things get back to normal, we adapt to the crisis. So that was very interesting. We adapt to the crisis, in other words, we modify the company's organization during this phase by breaking down a lot of barriers.** On the other hand, the smart thing is that as soon as things are more or less back to normal, we go back to normal organization. Because otherwise, it's too much to manage, because it relies on different people who have to do their jobs on top of that. So we know what we have to adapt to, depending on the Covid enemy we're up against.”

**Interviewee:** Okay. And are there any innovations that have come out of the crisis for you?

**“Somewhere, again, and I think that the relationships that were established during these white plans etc., the people who were revealed or who took charge of things and for whom it was successful. People don't forget, they remember. With occupational medicine, we collaborated very well with the ISD, with the nursing department, CV etc. So there you have it. So there you have it. So after that, there are links which mean that it's not necessarily friendship. We know people a lot better than we did before, and what's more, we've worked in conditions that weren't, weren't normal working conditions. So that means we help each other out. You do this, I do that, so we're all in it together.”**

| Quote from hospital director about the interdisciplinary coordination during the vaccination campaign |
| --- |

When the vaccines arrived, we made sure that all practitioners and carers could have immediate access to the vaccine, because we had been given the impression that the vaccinated and carers had protected themselves from the patients, obviously. Organizing an on-site vaccination center for patients who wished to benefit from vaccines, we had a really voluminous action in terms of vaccinating both patients and carers. Thanks to a remarkable occupational health service, and employees who had retired from the center who were able to come back and help us vaccinate everyone.

1. **Transferring patients from a hospital service overburdened due to COVID cases/creation of new hospital departments**

| **Quote from neuro-oncologist on creation of new hospital department for COVID** |
| --- |

“We have multidisciplinary departments, so we have up to 175 Covid beds, so we've created an oncology department that was isolated. The hematology department was untouched, and the dermatology department, which does a lot of melanoma, was also preserved: half the melanoma beds, half the non-Covid general medicine beds. And we've created - well, we created it for me, because I'm the one who took over this unit. Fourteen pneumo-cancer beds. But with nurses coming from endocrinology, with interns coming from endocrinology or elsewhere. **It's a Spanish army, but it worked very well.** But the patients...we saw lung cancer patients arrive at the last stage of life directly in the ICU, we in Lyon, since we were the hospital that took the most Covids...but at the time we had, we held a meeting to decide, because there, the ICUs were full to bursting and we had said to ourselves how do we determine the age of patients who go to ICU having or comorbidities. **But the wave stopped. We had a decline just when we thought we had to make choices**. Okay, we didn't need to make any choices. Patients who had to be resuscitated, saying "I'm not resuscitating this one", when there was no associated illness.”

| **Quote from neuro-oncologist on creation of hubs** |
| --- |

“Perfect transparency....perfect solidarity. So yes, it's been good. **We made hub systems, so we had a cell. When we thought a patient could go out, you can't go home yet. There were people who found beds in clinics. From right to left.** It was a Covid cell, but there were, it was by establishment...South, they were different hospitals, different clinics than the East or the North. We have a total solidarity that our colleagues don't necessarily have, and then we had...the internal medicine service stopped the endocro service, the rheumatology service and geriatrics. Everyone made savings. And the gastro service too. There's a surgery department that's become Covid surgery, so it's 75 beds, and SC has become the person in charge, so we've doubled the shifts, so there are two people on call for Covid people, and one non-Covid person on call.”

1. **COVID reflection cells**

| Description of the role of COVID committees in the private hospital (pseudonym is identified as Chloé in the article) |
| --- |

“And my teams were exhausted. I mean, I felt like I was putting them...well, questions and answers, questions and answers. But I felt I had to be or assume leadership there. So he said, **"We're going to team up. And at that point, the two of us got together and we did the briefs, and at that point we set up the Covid committee every evening at 5 p.m. and everything I'd done during the day, we debriefed and asked questions. And then some doctors joined the Covid committee, me, hygiene, and we said who could answer and we set up an e-mail address where we could ask all our questions. And that team, from seven in the evening to 5 p.m., we set up this Covid committee, took minutes and briefed our teams in the morning, and we structured it. At first, it was very much a care team, and then we set up medical, care, pharmacist and logistics teams. And that was because everyone was in their own corner, because they didn't know at first. But then, we got into the swing of things very quickly, and that gave us the impression that this was it. We structured ourselves and did things together. There was no more ego, and that was really extraordinary.**

| Description from hospital director on the role of COVID committees in the private hospital |
| --- |

Interviewer**:** can you tell me a little bit about the Covid committees...how you worked together?

Hospital Director, “We created it (long pause)...doctors, nurses, pharmacists, technical staff, administrative staff. It worked on a daily basis to answer urgent questions and interact with the management committee. It made proposals for both communication and organization in virtually real time, enabling us to respond to the crisis cells that could emerge when we had crises of access to medicines, or to the various different tools that enabled us to manage things. The Covid committee's work themes varied: staff protection, patient access, welcoming foreign patients, organizing and coping with the influx of patients and reorganizing services, specific on-call duty. These are just some of the topics discussed by this multidisciplinary committee.

**That's what collective decisions are all about, feedback from the field. It's really important for caregivers and administrators to know what they're up against, so they can adapt. It's quite amazing to see how much discussion is needed at these meetings, because everyone has information from different sources.”**

1. **Limits on interdisciplinary coordination**

| Quote from vice director on limits of interdisciplinary coordination |
| --- |

“No, I think the major difficulty during this period is the coordination between the hospital, because each hospital takes the decision for themselves, and then there is no coordination for example, we don't perform a hip prosthesis, there is no hip prosthesis during several months. And that's a problem now, because there is a lot of people to need the prosthesis. Perhaps we have to choose one hospital for orthopedics, for functional orthopedics and all the surgeon all the people could have surgery in this hospital. For us, we are specialized in cancerology, perhaps we could receive here some people from other hospitals with surgeons, with...I don't know, to focus on cancer and treat the patient for cancer only for cancer and for this is the same for all...and for example, the HCL could treat the patient with COVID. **This is a decision above us, and there is no decision of this type of this kind of decision there is all the hospitals are all alone. We decide we decide for ourselves.** All the guidelines, we decide for organization of the trials, that's a good way but why I don't treat the patient with breast cancer who are delayed in another hospital? Just my neighbor here N, that delayed some patients with cancer. Why we don't do here? We prioritize patients with cancer to treat…to make the best treatment for each patient with more capabilities. **I think this is the major lack during the crisis. Because all the people organize themselves. All the hospitals, we are some capabilities to answer to the crisis. But there is no coordination between all the health organizations in the metropole.**

Interviewer: Who could do that, the ARS (regional health authority?))

Healthcare provider, “I don't know ARS...ARS delegate to the public hospitals, but this is not the good way because the ARS could not delegate this this kind of decision at the hospital...but in my opinion, this is the most important thing to do. **In case of a large crisis like the COVID. We have to organize differently all the organization of the health, of the health organization. And to choose or not, I don't know. But we have to, to have a reflection at a different level, the good level is not the hospital. This is the region. Or the region but this is not just the hospital.** I'm a little bit idealist, but this is because the income of the hospitals are calculated on the activity. So each hospital wants to keep their whole activity even during the crisis. That but during the crisis, we have to transcend that. And to have a large region. But each hospital wants to keep the patient because we found that if the patient go outside or go in the other hospital, they stay there. I think that's the reason.”

1. **Research on COVID**

| Full description by clinical scientist at public hospital on interdisciplinary work in COVID research and how the crisis helped them to better organize research across sites (identified as Martin in the interviewees) |
| --- |

“So, what I'll remember the most, because I'm sure I won't be able to remember everything here, what was very, very complicated to manage at the beginning, was the almost total cessation of most studies, because we had to mobilize beds and entire departments to receive Covid-positive patients. So, at the time, there was a message from the DRS, which was still called DRC at the time, to the effect that research activities had to be stopped, which was not necessarily well received by all clinicians. But then...the second milestone was the implementation of the Discovery trial. I don't know if others have told you about it. It was a European trial aimed at finding a treatment that was piloted by FA. It was a European trial with several treatment arms to find an effective treatment for Covid as quickly as possible. And that was quite complicated to set up because, in fact, it involved ICU patients who changed departments once they were no longer terminal... we had to find CRAs, we had to mobilize people from certain departments who were no longer active in other departments to come and help out, which was greatly impacted by Covid. **So we had to convince the doctors that their staff would be more useful elsewhere. Mobilizing these people. That's what really impressed me at the start.** All the administrative upheaval, that's what brought us to light. I'll call them shortcomings, but maybe that's not the right word, but it highlighted a few points where we weren't necessarily very good and on which we've been working ever since.

To be perfectly honest, research at (the public hospital) is something that's lacking. A lot of work has been done over the last few years, but historically it was quite...how can I put it? It wasn't that it wasn't considered by the institution, but it was organized in such a way that clinicians organized themselves, went out and got their own funding, recruited their own ARCs. It was a bit obscure for central management and we don't have any research support structures like CECs and CRCs, which is also why I'm here. I was recruited for this position and so, in fact, here we're a residential hospital, so there's a bit of research going on all over the place, but people don't really know what the others are doing. And then there are assistant research coordinators everywhere, but they don't necessarily know each other. In fact, it's just like when we had to make a census of people, particularly to find out who could be redeployed. Well, it wasn't necessarily that simple, because we realized...not me in my group, because I had done the mapping, the census, the inventory. But in other groups, we realized that we didn't have an exhaustive vision of all the research staff present and that **we needed to redeploy these staff correctly and efficiently in the departments that needed them most, i.e. the ICU, internal medicine in emergencies, to manage Discovery-type trials in particular, but also other trials that arrived in the first wave to find a treatment quickly. Well, it wasn't always easy. So, this observation was made, and since then, we've been working on structuring and getting to know better the agents who work in research. That's all there is to it.”**

“We've done it on several levels. More recently, our new director has restructured our research department and created an HR department. In fact, because until now, we'll say that research, the research department managed research more from a budgetary and financial point of view, and less from an HR point of view. And it's typically the kind of activity that's a bit between two waters at the hospital, between two departments, without really being taken on by either one. Some things were done by the local personnel office, but not everything. As a result, the creation of this HR department is intended to identify, list and establish self-updating mailing lists. All research staff working at the hospital. So that we have a good knowledge of all the professions that exist. So there's a whole HR policy that's currently being reworked. We're **redoing all the job descriptions, we're reworking all the missions, we're doing this work. We're also getting to know all our staff. It also means, for example, that if someone wants to change someone of value, we want to keep them. It allows us to offer other positions in other groups to avoid losing this kind of thing. And that's it. So I think it's served us well. This first wave of Covid made us realize that we didn't have an exhaustive view, particularly of our research staff.”**

“The situation stabilized fairly quickly for research, unlike care, where we saw them struggling a bit, skating along, undoing and redoing three months later, remobilizing the services to put them Covid more. We also saw the climate, the climate, on the sets. Here, there's a plateau of consultations and ages of life, the difficulty of setting up double circuits, but research after the first wave, research has been less impacted, at least in terms of organization. Well, the big Discovery trials had been set up. That was the hardest part. **In the end, it was the first wave, the setting up of these large transverse trials that cost several teams, from several groups in particular, since these trials were deployed in the south, they were deployed in all the groups. So we had to coordinate. It wasn't easy, not everyone had the same working methods, and so the research project managers in the field, like me, there aren't any like me anywhere else, but there are some in support structures like the IARC, and so it was the project managers with our directors, of course, who coordinated all this to ensure that we came up with something that held together at the other end and that everything worked well.”**

- - 1. **Telemedicine and teleconference technologies**

| Quote from hospital manager in private hospital about pre COVID vs post-COVID era in terms of teleconsultations |
| --- |

“So I think that, X told you the figures, but I always like to remind people of them, eight weeks before the first lockdown, the giant. **We had done 25 teleconsultations. Eight weeks after the Giant of the first containment, we had done 5,584 teleconsultations. I think we said it all when we said that...it's something that's been maintained. In other words, even today, around 20 to 25% of consultations in the provinces are teleconsultations.** This has enabled us to meet the needs of our patients, but also of patients who come to us for treatment. And there are some**. It's almost paradoxical what I'm about to tell you, but we've observed that many patients find it easier to contact us nationally and even beyond, in Europe in particular, and to contact us personally....**I'm thinking of a French patient who lived in London, who couldn't be treated in London hospitals for various reasons, and whom I was able to follow up and treat in consultation without ever having seen him, until the situation became normal. **So these are fairly exceptional situations, but they have been made possible by teleconsultations, and have transformed our practice, since it has now become common practice.** The same goes for multidisciplinary consultation meetings. As you are well aware, these are at the heart of our action, and for a long time they were largely virtual, with only two or three people present, but now they remain partly virtual for those who don't want to or can't travel. So these adaptations have had their positive aspects.”

| **Temporality/space** | **Value for the patient/healthcare provider** | **Temporalities/spaces where it was not recommended** |
| --- | --- | --- |
| Pharmacy | Renewal of medication; patients lack time or cannot come in person; when patients need an additional explanation about medication. | First prescription with a patient |
| Post-procedure (surgery) | Keep the link with the patient before the next visit; permits healthcare provider to explain the surgical procedure when patient is more ready to receive information (at home, post-surgery) | Bad news |
| Communication about diagnosis | Announcing “good news” such as cancer remission | Breaking bad news |
| Patient/professional in their own home | Reduction of stress/burn-out both for the healthcare provider and the patient due to time gained | First consultation; new treatment |
| Care for fragile patients | Vulnerable and/or elderly patients living far from hospital (who do not need necessarily to come to the hospital) | Patients with few/low internet capacities or connections |
| Territorial inequalities | Increased access to patients that live far away, in particular from national reference centers and to bring care access to those who are out of the country/region | For those who can travel to the hospital site, most patients/healthcare providers continued to prefer in-presence care |
| Teleconsultation to inform patients of clinical trials and get electronic consent for participation | Helped continue research in spite of sanitary restrictions | May be used for future sanitary crises, but not ideal in normal clinical research settings |

1. **Pharmacy**

| **Pharmacist description at public hospital in the instauration of different pharmacy care pathways** |
| --- |

“A great deal of mobilization on the part of the team, with the promotion of the work that had been done, with communication on the networks, photos and then the support of the public, which came to us in various forms and which, I think, was very important. That's it for the oncology part, again for the oncology part, so a lot of work has been done at the level of the French Society of Oncology Pharmacy, with a survey of practices and an adaptation of manufacturing procedures, hygiene and so on. Work in cleanrooms based on Covid, i.e. on the model of what has been done at Lyon Sud, i.e. to ensure that all procedures are respected in the manufacturing room and then to set up procedures outside the manufacturing room, outside the controlled atmosphere zone, and then to ensure the link between the two. Still on the SFPO side...the coordination of a position paper on the management of cancer patients during the pandemic. So here we are, and the SFPO has mobilized and produced both practice recommendations on the manufacture of injectable anticancer drugs and a position paper on the management of patients on the ruptures on the problem of continuity of care. **So we have a major program called ONCORAL, which monitors patients taking oral anticancer drugs on an outpatient basis. As a result of the reduction in the number of outpatient visits, we've developed remote medical consultations, and we've also developed remote monitoring of pharmaceutical patients, i.e. we only see patients who are on the move. In fact, we've always only seen patients who came in for physical consultations, and as these patients were often seen in remote consultations. So for these patients, we adapted a remote pharmaceutical and nursing consultation as well. This enabled us to work on our ISILI tool, which allows us to see patients remotely. It also allowed us to think about how we can convey information to patients when we're working remotely. Because we're not handling the medication box and so on to explain the treatment plan, things like that. It also enabled us to measure the proportion of patients who might be receptive or well-equipped for remote consultations, so it's not insignificant**. **So we switched to remote consultations for patients benefiting from clinical trial treatments, something we'd never done before. We sent the treatments either to the patient or to the pharmacy. This meant that we had to set up a network to ensure that patients benefiting from a cancer clinical trial would receive their treatment, even if they didn't go to the hospital pharmacy, to avoid exposure. Because, as in all other establishments that treat cancer patients, we obviously took into account the fact that patients would not be exposed to the risk of exposure. We had obviously considered that cancer patients were a risk, and that if we could really limit travel to the hospital, it would be better for them, especially at the beginning, especially in the first wave, when we had little information about what was really going on, how the virus was being transmitted, the fallout, etc. And before vaccination. And before vaccination. So we got in touch, we organized with the agreement of the promoting pharmaceutical laboratories. So it's no mean feat to send clinical trial drugs to the patient's home or via pharmacies. And I know that this is something that has been done by other teams.** In France, we're not the only ones, but I think it was an interesting way of ensuring continuity. So, in fact, we ensured continuity both for the follow-up of patients undergoing oral chemotherapy through consultations. We ensured continuity by referring patients for treatment as part of clinical trials. And I'd say we've continued to support the departments in the preparation of injectable anticancer drugs whenever possible, i.e. when the patients were there, they were all treated. Now, we're all aware that there's probably a reduction in screening diagnoses, and probably in treatments too, during this crisis. During this crisis, I believe it was evaluated by various bodies. **There are publications on this, so it's very clear. I think that, as pharmacists, we haven't been blockers, we've been more in the role of support and more in the role of making proposals to avoid disruptions in care.** So that's it. Overall, I'd say that's what we've done for oncology. The pharmacy team was involved outside the cancer ward.

1. **Post-procedure**

**Quote from endoscopist about how improving communication post-procedure was made possible via teleconferencing technologies**

“It's back to normal, it's back to normal. The only difference is that, ideally, you'd be a doctor with lots of time on your hands and patients in hospital. That's my ideal. That's not the real world. Which means you go to see the patient the day before, to check on him, to see how he's doing, you send him back after his procedure, to see if everything's okay and let him go, and the patient is in a comfortable hospital, well, that's not it anymore. Now, the patient is in a day hospital, he comes from far away, he arrives, he leaves. **You have time to see him after the procedure, when he's in the recovery room, not fully awake, not perfect because he's just had anesthesia, and you explain things to him. If he's in the hospital, you don't see him the day before, you don't see him the day after, so you see him after the procedure, it's not the same, it's not the same doctor-patient relationship. It's not the same, it's not the same doctor-patient relationship. These are modern working methods that are moving fast, and we're going to have to find improvements and improvements. For me now, it's all about trying to see the patient again in a remote consultation to explain the procedure. I'm doing it more and more because the hospital system means that we no longer have the time to do that. It's not, it's not good, it's not for the benefit of the patient or the family.”**

1. **Communication about diagnosis**

| Quote from endoscopist at the public hospital about communicating a diagnosis by teleconsultation |
| --- |

“At the end, some things will stay. You're talking about cancer. Cancer is very special. It's called a consultation d'annonce... obviously, it's done face to face, not by teleconsultation. It's absolutely horrible, but horrible or not. Maybe one day we'll get there, but in any case, it's not done today. It's not moral, it's not adapted. I do a lot of genetics and I go to genetic diagnosis consultations every day. That's it, and that's all there is to it. I don't think there's anything. It's even worse than a cancer diagnosis, I think. Because you've got the whole family, you've got the parents, you've got the guilt, you've got the siblings. The diagnosis of a genetic risk. You've got four siblings. Two are at risk, two are not. Come and see a consultation like that one day, and you'll see how much electricity and emotion there is in the air. That's what it takes. Obviously, it's not going to happen at a distance. It's impalpable at a distance.”

1. **Patient-professional in their own home**

**Quote from gynecologist about how their service was impacted during the crisis and the positive impact of teleconsultation for both the patient and the healthcare provider**

“In fact, we were very spared by Covid because we had a large maternity unit and an emergency department. We kept our operating theatres open because we were obliged to provide continuous care. As a result, we were able to continue all our oncology work without too much difficulty. So we were very lucky. We're not so lucky today, because we're short of staff. A more difficult configuration today than it was during the Covid, because we're an emergency door service. And we also didn't have any Covid in-patients on our premises, so we kept our health service. So we didn't have a year. We weren't really impacted by Covid, even the staff, there were very few who had to go elsewhere because we had an activity that was maintained afterwards. The mother and child hospital, we have a separate gynecology unit that's coupled with the maternity unit, with a shared team. So when the maternity unit was out of the question, when it stopped, we didn't maintain our technical platform and emergency services, because we have a large emergency department. So I think we lost a lot of chances for patients who didn't come for consultations. But for those who were in the active care queue, we took care of them. **But the fact that we also did a lot of video consultations. We tried to keep the link and that's what we kept. After that, it's great, I had a day off, half a day with visioconsultations and that's great. The quality of the work. We get half a day away from the hospice phone and all that, and we're on video conferencing for the patients, and it's very pleasant….for the patients, they're very happy not to have to travel too. Biopsy results for suspected cervical lesions, abnormal smears. We suggest, we give the biopsy analysis. You're told to have a smear or a test in six months. You don't have to go to a consultation to give them this kind of information.**

You have to select the files with which you're going to do the visioconsultation. You can't do visioconsultation on everything. After that, it can also be intermediate consultations. To keep in touch with patients, to maintain an active thread, a link. A more important link via the hospital, because it doesn't necessarily take up too much time. And it can also be a way of keeping in touch with patients or exchanging ideas. In the immediate aftermath of a procedure or surgery. Even if you have a post-operative visit scheduled several weeks later, it's the quality of the quality, of the follow-up and. And that's it.

**Interviewer:** And I'm just curious. Does it? Do you think it evens things out a bit between caregivers and patients?

**Healthcare provider:** Yes yes, they are, they're more comfortable. And then they're more relaxed. They're not going to run for parking, take the kids fast to school to be on time for the appointment. After that, it's not for all patients either, but for autonomous patients, patients who are at least comfortable with technology.

1. **Care for fragile patients and territorial inequalities**

| Quote from endoscopist about caring for fragile patients by teleconsultation |
| --- |

Interviewer: “Did you use teleconsultation during the crisis?”

Healthcare provider: “that's one positive thing that came out of the crisis, and that's that I now do a lot of teleconsulting. But in fact, I've done it because it's very useful for two situations. **I see people who are in the region, elderly, frail, who don't need to travel for a consultation, by teleconsultation.** Secondly, we're a referral center. So I give consultations to people in Nantes, Brest, Strasbourg, etc. And it's obvious that this had to happen. It's just so bizarre that we hadn't done this before. Three years ago, people were coming to me from all over France for a consultation. I don't need to see them for most of the things I see, what I want to do with them, I just need to see them on a screen and explain things to them. It's just as effective for that. That's the computerization of communications, of meetings... we could talk about that. Computerized meetings are a huge thing. And teleconsultation is a huge thing.”

| Quote from hospital director about reducing territorial inequalities by teleconsultation |
| --- |

“It's almost paradoxical what I'm about to tell you, but we've observed that many patients find it easier to contact us nationally and even beyond, in Europe in particular, and to contact us personally....I'm thinking of a French patient who lived in London, who couldn't be treated in London hospitals for various reasons, and whom I was able to follow up and treat in consultation without ever having seen him, until the situation became normal. **So these are fairly exceptional situations, but they have been made possible by teleconsultations, and have transformed our practice, since it has now become common practice.** The same goes for multidisciplinary consultation meetings. As you are well aware, these are at the heart of our action, and for a long time they were largely virtual, with only two or three people present, but now they remain partly virtual for those who don't want to or can't travel. So these adaptations have had their positive aspects.”

1. **Teleconsultation to inform patients of clinical trials and get electronic consent for participation**

| **Full quote from head of research in private hospital explaining about new methods for clinical trials during the COVID crisis** |
| --- |

“So basically in the Covid epidemic, basically, we are really impacted by the first step of COVID. And it was a real, a true difficulty to, to take charge of patients in clinical study, to obtain new clinical studies, to follow the patients included in the clinical studies, clinical trials. And so during…I don't remember exactly what how many weeks the first steps but I think it was two months? **And during this first two months, we had to fix a new organization to take charge of patients.** So, we had several difficulties. The first was to give the treatments from our pharmacy in the hospital, from the institution to the patients, because we have many oral treatments, and targeted therapy, for example. And so basically the patient goes to see the pharmacist here in the hospital to have the treatment because it's not possible to have this treatment in the city in the classical pharmacy**. So, we have to organize a new schedule, a new organization, to deliver the treatment to the patients and to send directly the treatment to the patients. And it was new because basically the organization and the different recommendations for this did not anticipate this case. So we have to we had to work with competent authorities for this and we organized with a pharmacy some schedule…yes, we use some schedule to send the treatment at home for the patients.**

After it was difficult. Basically, in the clinical trials, we need to see in the consultation, at the hospital, the patients within a very strict interval of time. For example, every six weeks or four weeks, or eight weeks, two months, and the patients are to have a for example TDM, imagery, CT scan. **And it was very difficult to try to organize this. And so we need to organize the follow-up of the patients with teleconsultation.** Basically, outside the clinical trials, it was the same, I think, for the clinician. They had to continue to see and to follow the patients but in clinical trials, it was the same. So we organized some teleconsultation for this**. After in a classic clinical trial, we have a team of clinical research assistants, and they organize the follow-up for the patients during the trials. A**nd for example, they give some documents, they give some consent form at the beginning, they provide other quality of life advice, etc. So especially in this institution, we have a solid connection between the clinical research assistant and the patients included in the clinical trials. With the Covid-19, it was not possible. So we need to organize these kind of things by phone or video conferencing, so it was difficult, yes. Because the patient, we have many, many, many questions. It wasn’t safe for the patient to go in the institution. It was a total overhaul during the total lockdown, so it was a maybe it was not safe for the patient to have normal interaction with our team in clinical research, with the doctor but not only, with the clinical research assistance. And so it was the logistical aspect of the pandemic.

The other side of the problem was we did not know very well the disease. It was new for us, it was new for every clinician in oncology, like it was a was a new field of research. So we had the question: maybe it's not safe to give experimental treatments to our patients with the possibility to have the COVID-19 disease shown at the same time. So we have to stop anyway. So we decide and because we are sponsors of clinical trials, we have our own clinical trials, and it was the same decision for external sponsors, especially industrial big pharma sponsors, to stop the inclusion of new patients in clinical trials and to postpone new clinical trials in our institution or in different hospitals in France in oncology. So no new patients is already open clinical trials and no new clinical trials. So it was during the two months, it was really a stop in the activity of clinical trials in France. So this is maybe the two different sides of the problem. First, the logistics needed to reorganize and secondly, maybe it's not safe for the patient to give experimental treatment…immunotherapy, targeted therapy, chemotherapy, because maybe they will have Covid-19 at the same time, maybe we will have an interaction between the virus and the treatments and maybe it will not be safe for the patients. Yes, I don’t know if this is clear, I tried to remember the situation. It was totally new, in all the fields of society, we have to organize this in a few days, one weeks, so the first, two, three weeks were very (pff) strong yes. A little bit stressful.

So say after day, week after week, we learn about the situation. And gain knowledge and skills about Covid-19. So because of this, because we had more papers, more bibliographical abilities, we understood that maybe we can include clinical patients in clinical trials maybe it’s not a problem to have the disease and experiments and how patients need to have it…especially if you have a disease they need to continue the classical treatment as they need to have to be…yes we need to take charge of the patient, basically with efficient treatment. So we don’t need to stop all the inclusion, we need to continue to give innovative treatments to our patients. **So it was a change in paradigm**. So for example, in the second step of the pandemic, I don't remember that we have a bigger, big slow…and after I don't remember maybe after that, we have the second wave, so for the second step of this pandemic, because we learn, we had a better vision in how to manage the patient in clinical trials. It's possible to continue to include patients in clinical trials, it is possible to give innovative, experimental treatments to our patients, it is possible to open new studies. Yes, yes. And basically it was a little bit like the life before. Yes, yes. Yes. So the first step was bigger. It had a big impact to our organization because we didn't know how to do it, with the possibility to have adverse events between the virus and our treatments. But after we learn, and the second step…we need to organize a little bit the work of the team because yes to sometimes to have patients, the patient, the clinical research assistant, it's possible to work at home maybe one day or two days a week, after it was basically like the situation before the first. So there was a big difference between the first and second step for the clinical trials, yes. Not maybe not very, for the organization of the classical care for the patients, but for clinical trials it was a little bit the same, yes.

It was the difficultly that in France, and in Europe, we have very strict, strong for the regulatory aspect, it was…when you cannot do what you want, exactly what you want, even if you have this problem, this pandemic. So we, it was possible…first, to give the treatment at home, after organize the monitoring, the monitoring it is we need, you know, in clinical trials to control the quality of the facilities. And we need basically we need to, if you are a sponsor for clinical trials, you need to send clinical research assistants to control the activity of the clinical research assistant in the hospital. So, it is the same name – clinical research assistants – for two different tasks. You have in the institutions the clinical research assistant who will put the data into the form. And you have the second type of clinical research assistant to control to verify that the data of in the case of a form are the same as into medical data of electronic records. **So it was possible to during this first step and to discuss with the competent authority to organize the monitoring not directly in the hospital. But that's a special connection, the Clinical Assistant needs to be able to control at home, needs to have a special access of electronic record files for the patients. And it was new…we need an authorization from the CNIL. And we had a special authorization during the pandemic, now it’s finished…they stopped, I think the beginning of this year but it was specific to organize like this, it was possible to give if we need to include a new patient, in a new clinical trial, to give informed consent not directly to the hospital but to send by email, in order to have electronic validation by the patients. Yes, I want to be included in in the clinical trial. I give my authorization with electronic signature. It was new, because before it was not possible to do this.**

Normally, basically, the most important when you explain your clinical studies to a patient is the interaction between the doctor and the patient. Because the patients trust the doctor, you need to have a special interaction with the doctor. And this is an more important part and after the clinical research assistant in charge of the studies can give additional information to the patients. More logistic information. **So we need to organize this first, for the doctor by teleconsultation. Yes. And after by conference call with a clinical research assistant to give additional information to the patients, what logistics, in particular, what would be clinical studies in your life…so, it was a little bit different…we send the documents from the studies to the patients by email. And the patients have one to three days to answer and after to give the informed consent by electronic signature.** But the two steps. First, the more, most important, is the information that the doctor could give by teleconsultation or in the hospital when it’s possible of course, but after the additional information by a special conf calls and by email, we have a special application, a special file , you can have specific information about your case. So it was possible to have some extra discussion by this way for example. So it was a it was, what is possible, of course, at the hospital…what is not possible, we have the possibility during the pandemic to send the documents at home for the patients, and to have interaction by teleconsultation or by phone or by email with the patient….normally if you are a patient, you can talk to your GP, yes, do you think it’s good for me? You can ask your family, you can talk with your wife, your husband...of course it is like that. **Now it was a little bit different. Because always by electronic way…yes, normally it is easier for the patient to have direct interaction between the teams between the doctors, the clinical research team.”**

Interviewer: now we're saying that teleconsultation can be a good solution for (DP: yes), for many, but in the case of clinical trials, is it adapted?

**“Yes, for me yes, it is a good possibility/alternative option. But in the other case the patient needs to see in the hospital.** So I think it depends of the possibility for the doctor to have a good interaction at home or not for the patient. We have many I think we had different levels, different situations, but for that you need to ask directly to that to XX. Because for the trials it was maybe a little bit difficult. But for the first time we had the authorization from competent authorities, from the regulatory boards, to give informed consent by this way, to do teleconsultation…so it was new, before all was in the hospital in consultation with the doctor, time with the clinical research assistant, you have time at home to think about this. So you have time to give your decision. Now it was during the COVID it was a little bit different. But I think we had this possibility during the first step. And after I think, the possibilities were always open. Now I think the situation is now like before.”

**3.1.4 Creativities in the care plan**

| Description of the creativities in the care plan (pseudonym is identified as Chloé in the article) |
| --- |

**“People have gone back to the source. Why are they doing the job? And I've never seen so many caregivers come up with creativity.** And just before you, my dear M started drawing and making comics for patients to explain the disease. Because she realizes that in fact...everyone is back to saying OK, what's important to me? And it's true that Covid came to question. What was so important to me? Because life or because we also realized that we weren't protected...in fact this feeling of insecurity, it's that ok. But what makes me feel secure? And is that where I come in? But really, sincerely, during the whole confinement, we had to write 450 pages of how to take care of the premises for the patients, how to take care of the patients? **And we wrote everything for a year, made comics, newspapers, there was one who did drawings with the patients and exhibited the drawings...one who started making music. They brought in a lot of instruments to give to the patients. I don't know. There's a lot of creativity, a lot of questions about pets. They'd like us to work on how to ensure that patients who are good, who are hospitalized for a long time, can be reassured about how their pets are looked after and what they can see of them. Is this the question that's never been asked.** **Executives and managers are questioning their place and the way they operate. What's the right thing to say, what are the right values, and what are the values themselves? We've never questioned our values so much, why we do the job we do, what are the values we've been looking for together.** So there's more truth, I think... behind all that there's more truth, which is just as hard as it is pleasant. It does have that effect. It's amazing how truth makes people see the most beautiful and the most ugly. And I'm delighted to see how much is moving and how much talent is being released in our teams. Thinking about working hours. We're in the process of revisiting this. I've never accepted so many part-timers. We also want to give people time to be creative, to limit the amount of time they spend at work, to find other things to do, to give them the possibility of sabbaticals to find themselves. And that's where we now need managerial agility to hear that there's a new way of working together. And people want to think together. Really, they are and they want a vision and hope, of how we're going to make tomorrow that it doesn't take much for people on one side to be very, very sad about everything that's gone. And yet, new things are happening. Appointments...we can see each other again. We can have meals together again. What we experienced, what was very hard, was the lack of conviviality because they could no longer have coffee together. These are very basic things, but they're very important. So there you have it. I find that the release of creativity is so strong that it upsets me, because they're challenging me on what kind of manager I'm going to be tomorrow? Am I capable of hearing what they want? Am I able to be agile with them, and also feel upset by what I'm becoming? Because I, too, have found that I've changed because of all this. What's right for me now? And I can see that I'm changing too, that I'm also adapting my working hours. I'm changing the way I do things, so as we all change at the same time if we don't talk to each other in truth. The world will go mad. So since we're all changing at the same time, that's what brings us together. The common thread is change and the search for truth and hope. And I find that powerful. But it's going to be complicated all the same.

- 1. **Harmonization and streamlining of patient journeys**

1. **Harmonizing treatment decisions**

| Full quote on harmonization from a head and neck specialist (identified as Marie in the article) and description of how the specialty was affected |
| --- |

“So my answer will be probably divided into three sub answers. What about the patient? The patient load…number one? Did we second? Maybe not in the right order? patient load number first, for sure. Did we modify the processes in the two? And how did COVID crisis affect the MDs, the physicists, the medical physicists and radiographers. And maybe a forth parameter. There is always a plus even in a big crisis, in fact I think we got some pluses at least…I may be positive enough or naive enough, whatever you want to call it, to believe that we got some positive aspects.

2020, the first wave of COVID, we got the decrease in the overall activity of our Department of 3% percent. So in other words, we only treated 3% less patients then in 2019. Maybe it was two and a half. Yeah, it was maybe it was three, ya it was closer to three and a half. And in 2021, we did we recover the activity of 2019. So it means that in terms of the number of treatments, which is roughly a little below 4000 a year, we only decrease it by 3 and a half percent in 2020. And we recover it in 2021. And it's impossible to tell you what will happen in 2022, because we only have three months and we have we always have fluctuation which is absolutely unpredictable. For some reason with less patient in May or sometimes less in June, sometime we try to make prediction because of the vacation of the colleagues who refer the patient to us. But usually we cannot make any sort of explanation. So I don't know what will happen in 2022. But the numbers I'm giving you are 100% percent sure. Because every patient, every patient treated in this hospital, in this department, has a file in a particular program called Mosaic, which is the program that guides, and not only guides…pilots, all the treatment, so I know exactly how many patients were treated. So in other words, I can really predict up to the patient level.

So that's number one. So it means that at least at (the private hospital) **we cannot say that COVID impacted on our quality of treatments.** Now, the question is, number one, we are indeed, maybe more fortunate than others. And I know, one center in Roanne, I heard that they were for the first wave extremely impacted, really impacted a lot then decreased by 25 or even 30%. And this was because of the shortage of personnel. So probably a reason is that in 2020, we go to the second answer to my question…in 2020, no, MDs were affected, a physicist? maybe one? Maybe. We radiographers, so it was before vaccination, when the first 2020, I think. Maybe we lost one radiographer who was pregnant, by security and when the vaccine came, one refused to get vaccinated. And number three…and then one decided, so maybe one or two decided to move closer to the family location to be, to feel more secure. **So this probably explains why in 2020, we were really not affected. But maybe there was another explanation, which was we reviewed the processes**. So, when we got the information, early March 2020, then this was a big issue, potentially a traumatic crisis, that we might be strongly affected. Because, on the contrary, to a surgical operation, you operate one day, you open, close. That’s it. Full stop. For chemo, you inject one day, sometimes you infect few days, every month or something I let the idea is that when you start, you are ready for the next 3-4-5-6-7 weeks. So the issue was what will happen if in the middle of a seven week treatment with a shortage of MD, physicists, and we have to say guys, so you will never compete your treatment. This is absolutely impossible. So what we did in 2020, we do this in the process, I don't want to enter into too many details. **But number one, after discussion between us MDs, medical doctors, we agree to use a little bit more what we call hypo fractionation treatment. Slightly higher dose fractionation. The goal behind was at least the treatment time will be shorter.** And if there is a big issue to be, to deal with, to be dealt with, at least, we will be in a better situation to cope with these issues, too. That's number one..the things we did.

**Number two, that we did, we said we clearly need to harmonize the prescription of treatment among MDs. We know that MDs by definition and it’s good, have to have the freedom to decide what they do for their patient. Here we ended in a crisis and potentially a big and long crisis. So we need to refine things we need to say guys, sorry, no, let's do everybody the same.** **But depending on the urgency, depending on the complexity of the pathology, and so we divided the treatment into four categories: urgent, urgent, it's within 24 hours, bleeding, bleeding or spinal cord compressions…and on the opposite, we have the one with time, we know that if you have prostate cancer, you don't need to be treated in three days, there was an appointment, there is also a slow growing tumor, so this is when you know you’re three**. For number four, when you know if for post-op breast treatments, we know typically we have three months post-surgery, okay. Then, we have number two, which is pain. So parameters are that it is painful, easy treatment, we can start the treatment typically within five days. We don't, we cannot say we will start within 24 hours…no, no, because there are more things that can be delivered to patient but within short, five to 10 days and then the regular treatment, typically with in place, hypo-fractionation. So this was reviewed, by two of these, everyone (myself as head of department) and the vice head of department. **So every morning, we and plus a medical physicist, and a radiographer, we review all the requests by our colleagues, and we were “quote unquote” a little bit military people. Sorry, we are going to have a protocol for this…this is not 25 fractions, but because of COVID, it's only 15 fractions.** **We believe we did not put patients in danger. We strongly believe it. And by the way, everybody did the same.**

1. **Improved processes to better support patients**

| **Quote from endoscopist about improved processes to reduce hospital time** |
| --- |

“When you have, we play mainly because we do the care in the operating theatre. It's exactly the same. There are things that are or we aim for "best practice" best practices and best medical practices. And we don't change that. **What we have changed is essentially what we were lacking. There was a shortage of space in the operating room, so the procedures had to be carried out as quickly as possible.** So it was the interval between procedures that we tried to work on, whereas we were impacted by Covid on the contrary, because we had to disinfect the rooms. So that meant an even longer interval between gestures. But that's what we're in the process of changing. And above all, we've worked on the length of time or the way patients are hospitalized. Fifteen or 20 years ago, at the hospital, we had time. We would take people five days to do a colonoscopy. Right? Those were the days. The elderly patient who didn't feel like going home right away, who was a little tired, we kept. That was ridiculous. **Today, we do a lot of endoscopic surgery on an outpatient basis.** People arrive in the morning, they leave in the evening, we've gained in length of stay and we've found alternative means, especially to avoid blocking a hospital bed for three days. So people who come from far away. There's a system called the hospital hotel - they sleep there, they come to do the procedure, they go back to sleep with people who need a procedure that's not very dangerous, but who come from far away. **You see, in the hospital, to accommodate them, we do around the gesture, which is that we can now accommodate them in a system that is much less restrictive for the hospital and much less costly too, which is the hospital hotel.**

Now, the patient is in day hospital, he comes from far away, he arrives, he leaves. You have time to see him after the procedure, when he's in the recovery room, not fully awake, not perfect, because he's just had anesthesia, and you explain things to him, but it's not as good as what we used to do. If he's in the hospital, you don't see him the day before, you don't see him the day after, so you see him after the procedure, it's not the same, it's not the same doctor-patient relationship. It's not the same, it's not the same doctor-patient relationship. **These are modern working methods that are moving fast, and we're going to have to find improvements and improvements. For me now, it's all about trying to see the patient again in a remote consultation to explain the procedure. I'm doing it more and more because the hospital system means that we no longer have the time to do that.”**

| Full quote from gynecologist about transition to home care |
| --- |

(With home care),” we mobilize less risk of phlebitis, we feed ourselves better when we are at home. It also made us force anticipation and support. **The lady who is going to be hospitalization and who is going to go home in the evening, she cannot go to the pharmacy before. So we intensified the prescription of drugs in advance, she has to go to the pharmacy before the operation and so she can go home, she rests and she has what she needs at home. And also we call back the patients to find out if they are going…we accompany them**…for me, the COVID has brought us a lot in terms of organization and developing new organizations, video consultations, lightened care, yes.”

1. **Streamlining healthcare pathways**

| Full quote on streamlining healthcare pathways from a head and neck specialist (identified as Marie in the article) and description of how the specialty was affected: |
| --- |

**“The second thing we did also in the flow process, which I strongly believe it's a good decision, that's what I said the plus was.** So, this was all in one plus and the second one is that in the past, I see a patient in my clinic…I would ask the lady in charge of it, when could I start treating the patient without any categories? And she would tell me which machine do you need? Okay, take the particular machine first slot available in four weeks. Okay, so I go tell the patient, ok you will start the process in four weeks…the treatment process you start with is what we call the mobilization system, and then a planning CT, we will do the mobilization as soon as possible, do the planning and then wait until the patient slot is ready. Can be two weeks it can be for example, four weeks. So sometimes we're doing the CT and then one or two days after the request, the patient says, “sir, I decided I don't want your equipment and I want to go somewhere else closer to my place,” or in four weeks things may change. And in fact what you have prepared may not be acceptable. We said, this is a world for rich people…and we are not in a rich situation…**so we need to streamline this. Okay, so we said we just do the opposite. When do I have a slot? April the first…we'll do the CT two weeks in advance. And we know that the day you get the planning CT, you know it's mathematics. Two weeks after, you start your treatment. But before you get your planning, maybe it takes a week, maybe it takes two weeks, it may take a month if there is no real need.**

**So then you solve all your issues, but then the day the patient comes to the CT, you know, he agrees, and you will have his first treatment two weeks later, it's a different way of organizing it forced…the MDs to think twice before requesting a slot for the patient.** Yeah, maybe will not come because…I wait. No choice. And so it limited the lost slots. I don't know if you understand it may be difficult for me to explain it. **But it was for me really big plus in the COVID in the time of COVID crisis…we still apply it today. And I can tell you that it's exactly because I monitor everything. It's two weeks, from the beginning from the CT to the first treatment.** You may say yeah, but okay, you put your name on something. My name by the way? What's the point? **The point is that by limiting the lost requests, what we saw, it's not significant…but the time delay of recordings, between the time somebody’s asking, I see a patient in my clinic for radiation treatment. And the first fraction, overall, out of close to 4000 treatments, the time is slightly decreased by a few days. So in other words, by streamlining things, by really maximizing the slots that we booked for a particular patient, we minimize the losses and this was somehow macroscopically an advantage for department.**

So, I explained to you that we really changed the processes. The guidelines of the treatment standards, we are back to the normal standards today. We were lucky probably because we did not have sick people, at least in 2020. In 2021, so after the first and the second, and even the third now, vaccination, strangely but maybe not totally in fact, we got more patients sick, but they did not seem to be requiring hospitalization. They were out of for a week or less than a week, you know, the, the COVID measurement with the bit of child of fever and flu like symptoms. So, this explains why we only got two and a half percent drop in 2020. And then in 2021, we were able to recover everything. **So, I have to admit that the impact of COVID in the activity of this department were quite mild, if any. There was nothing magic.** Maybe we were a little bit looking 2020, not to have too many MDs and radiographers sick probably, because that's what I would like to call luck. Because, you know, we could have been in a party together and we could have been with 20 people sick the day after. You know, it is I don't know why is it so? **We did what the good sense I would like to call it ask us to do** and obviously it paid plus the regulation of this institution you know…replacement of this kind of meeting with you know, I’m now used to speak in front of the screen. I hate it, I still hate it…so that’s, in a nutshell…”

| **Full quote from endoscopist who was able to put into place streamlining procedures before the crisis** |
| --- |

“We have a great team of nurses and schedulers that, fortunately, I had put in place before the Covid crisis, because one of the department's problems was rescheduling and reconvening patients. We had set up a system, with extra staff, etc., which worked very well. It worked well and saved us a lot of money. But I think other departments had problems. We had really put in place, because it was one of my first jobs as head of department a few years ago, to solve this programming problem, and it saved us. Somehow, we were lucky, we had anticipated...without knowing it.”

1. VandeVusse A, Mueller J, Karcher S. Qualitative Data Sharing: Participant Understanding, Motivation, and Consent. *Qualitative Health Research*. 2022;32(1):182-191. doi:[10.1177/10497323211054058](https://doi.org/10.1177/10497323211054058) [↑](#footnote-ref-1)
